# Proposal for Human Respiratory Syncytial Virus Nomenclature below the Species Level

**DOI:** 10.3201/eid2706.204608

**Published:** 2021-06

**Authors:** Vahid Salimi, Mariana Viegas, Alfonsina Trento, Charles N. Agoti, Larry J. Anderson, Vasanthi Avadhanula, Justin Bahl, Louis Bont, J. Rodney Brister, Patricia A. Cane, Mónica Galiano, Barney S. Graham, Eneida L. Hatcher, Orienka Hellferscee, David M. Henke, Siddhivinayak Hirve, Sandra Jackson, Els Keyaerts, Leyla Kragten-Tabatabaie, Stephen Lindstrom, Inne Nauwelaers, D. James Nokes, Peter J. Openshaw, Teresa C. Peret, Pedro A. Piedra, Kaat Ramaekers, Annabel Rector, Nídia Sequeira Trovão, Anne von Gottberg, Maria Zambon, Wenqing Zhang, Thomas C. Williams, Ian G. Barr, Ursula J. Buchholz

**Affiliations:** Tehran University of Medical Sciences, Tehran, Iran (V. Salimi);; Hospital de Niños Dr. Ricardo Gutiérrez, Buenos Aires, Argentina (M. Viegas);; National Center of Biotechnology, Madrid, Spain (A. Trento);; KEMRI-Wellcome Trust Research Programme, Kilifi, Kenya (C.N. Agoti, D.J. Nokes);; Emory University, Atlanta, Georgia, USA (L.J. Anderson);; Baylor College of Medicine, Houston, Texas, USA (V. Avadhanula, D.M. Henke, P.A. Piedra);; University of Georgia, Athens, Georgia, USA (J. Bahl);; University Medical Center, Utrecht, the Netherlands (L. Bont);; National Center for Biotechnology Information, Bethesda, Maryland, USA (J.R. Brister, E.L. Hatcher);; Public Health England, London, UK (P.A. Cane, M. Galiano, I. Nauwelaers, M. Zambon);; Francis Crick Institute, London (M. Galiano);; National Institute of Allergy and Infectious Diseases, Bethesda (B.S. Graham, U.J. Buchholz);; National Institute for Communicable Diseases of the National Health Laboratory Service, Johannesburg, South Africa (O. Hellferscee, A. von Gottberg); W; orld Health Organization, Geneva, Switzerland (S. Hirve, S. Jackson, W. Zhang);; University Hospitals Leuven, Leuven, Belgium (E. Keyaerts);; ReSViNET Foundation, Zeist, the Netherlands (L. Kragten-Tabatabaie);; Julius Clinical, Zeist (L. Kragten-Tabatabaie);; Centers for Disease Control and Prevention, Atlanta (S. Lindstrom, T. Peret);; Imperial College London, London (I. Nauwelaers, P. Openshaw);; Rega Institute, Leuven (K. Ramaekers, A. Rector);; Fogarty International Center, Bethesda (N. Sequeira Trovão);; University of Edinburgh, Edinburgh, Scotland, UK (T.C. Williams);; Peter Doherty Institute for Infection & Immunity, Melbourne, Victoria, Australia (I. Barr)

**Keywords:** HRSV, Human orthopneumovirus, human respiratory syncytial virus, isolates, nomenclature, respiratory infections, specimens, strains, subspecies nomenclature, viruses

## Abstract

Human respiratory syncytial virus (HRSV) is the leading viral cause of serious pediatric respiratory disease, and lifelong reinfections are common. Its 2 major subgroups, A and B, exhibit some antigenic variability, enabling HRSV to circulate annually. Globally, research has increased the number of HRSV genomic sequences available. To ensure accurate molecular epidemiology analyses, we propose a uniform nomenclature for HRSV-positive samples and isolates, and HRSV sequences, namely: HRSV/subgroup identifier/geographic identifier/unique sequence identifier/year of sampling. We also propose a template for submitting associated metadata. Universal nomenclature would help researchers retrieve and analyze sequence data to better understand the evolution of this virus.

Human respiratory syncytial virus (HRSV) is the leading cause of severe respiratory illness in children <5 years of age and is associated with substantial illness from lower respiratory tract infections in industrialized countries and substantial illness and death in low- and middle-income countries (*1**–**5*). HRSV also causes severe disease among elderly and high-risk adults (*6*).

In 2016, HRSV was reclassified by the International Committee on Virus Taxonomy (ICTV) into a new family, *Pneumoviridae*, genus, *Orthopneumovirus*, and species, *Human orthopneumovirus*. (*7*). The wider availability of viral sequencing technologies has increased submissions of HRSV sequences to databases (Figure 1), a trend we anticipate will continue. Although ICTV provides nomenclature standards for virus taxa, there is currently no standardized format for HRSV nomenclature below the species level. Given the current interest in both HRSV and database submissions, a standard nomenclature is needed to simplify studies of the genomic diversity of HRSV strains and variants below the species level. ICTV’s taxonomic reassignment provides us a timely opportunity to propose a universal naming convention for HRSV strains, sequences, and isolates, including a framework for database submissions that are rich in contextual information and associated metadata.

**Figure 1:**
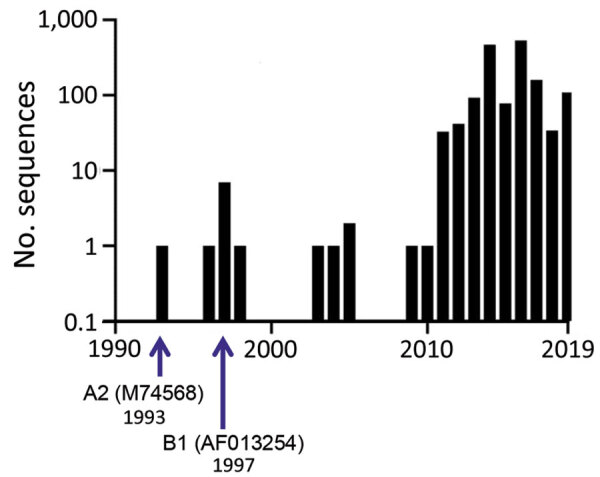
Annual numbers of HRSV whole-genome sequences released in GenBank since publication of the whole-genome sequence of HRSV A2, M74568, in 1993. HRSV, human respiratory syncytial virus.

Several large laboratory HRSV surveillance and epidemiology studies are currently in progress. These studies include the World Health Organization’s Global Respiratory Syncytial Virus (WHO RSV) Surveillance Project (https://www.who.int/influenza/rsv), which conducts large-scale testing for HRSV and extensive sequencing of HRSV-positive clinical specimens from >20 countries worldwide. Focused molecular analyses have helped elucidate HRSV household (*8*) and local (*9*) transmission dynamics and may guide development of strategies for the control of HRSV transmission. For example, molecular analysis showed that HRSV in healthcare facilities can be acquired from sources within the facility or introduced from the community (*10**,**11*).

In temperate climates, annual HRSV epidemics usually occur in winter months; it remains to be seen how social distancing measures and nonpharmaceutical interventions due to the current coronavirus disease (COVID-19) pandemic will affect global HRSV circulation patterns. One of the 2 major genetic and antigenic HRSV subgroups, A or B, usually predominates in alternating years, but both subgroups can also co-circulate in the same season. Early research has shown that subgroup A HRSV is associated with slightly greater clinical severity than subgroup B (*12*). Disease severity has been correlated with specific strains, genotypes, or clades, but to date, no consistent association has been established between strains (*13**–**15*), genotypes, or clades (*16**–**19*) and virulence. Thus, a possible role of different HRSV strains in disease severity remains to be elucidated. The lack of standard nomenclature and the scarcity of rich metadata in databases currently limit and complicate such studies.

Reliable and concise nomenclature systems below the species level are available for measles virus, influenza virus, rotavirus, filovirus isolates (*20**–**23*), and many other human viral pathogens. A similar nomenclature system tailored to HRSV and its pathology would support the requirements of researchers and the public health community by minimizing information errors when handling, storing, and shipping HRSV samples and when submitting, searching, and displaying sequencing data and associated metadata. Moreover, consistent nomenclature would improve the ability of researchers to pool and analyze data and associated information from different sources. To fill this need, an international group of researchers, in conjunction with the WHO RSV Global Surveillance Project, proposes a concise nomenclature system for HRSV below the species level.

## HRSV Genome Organization

**Figure 2:**
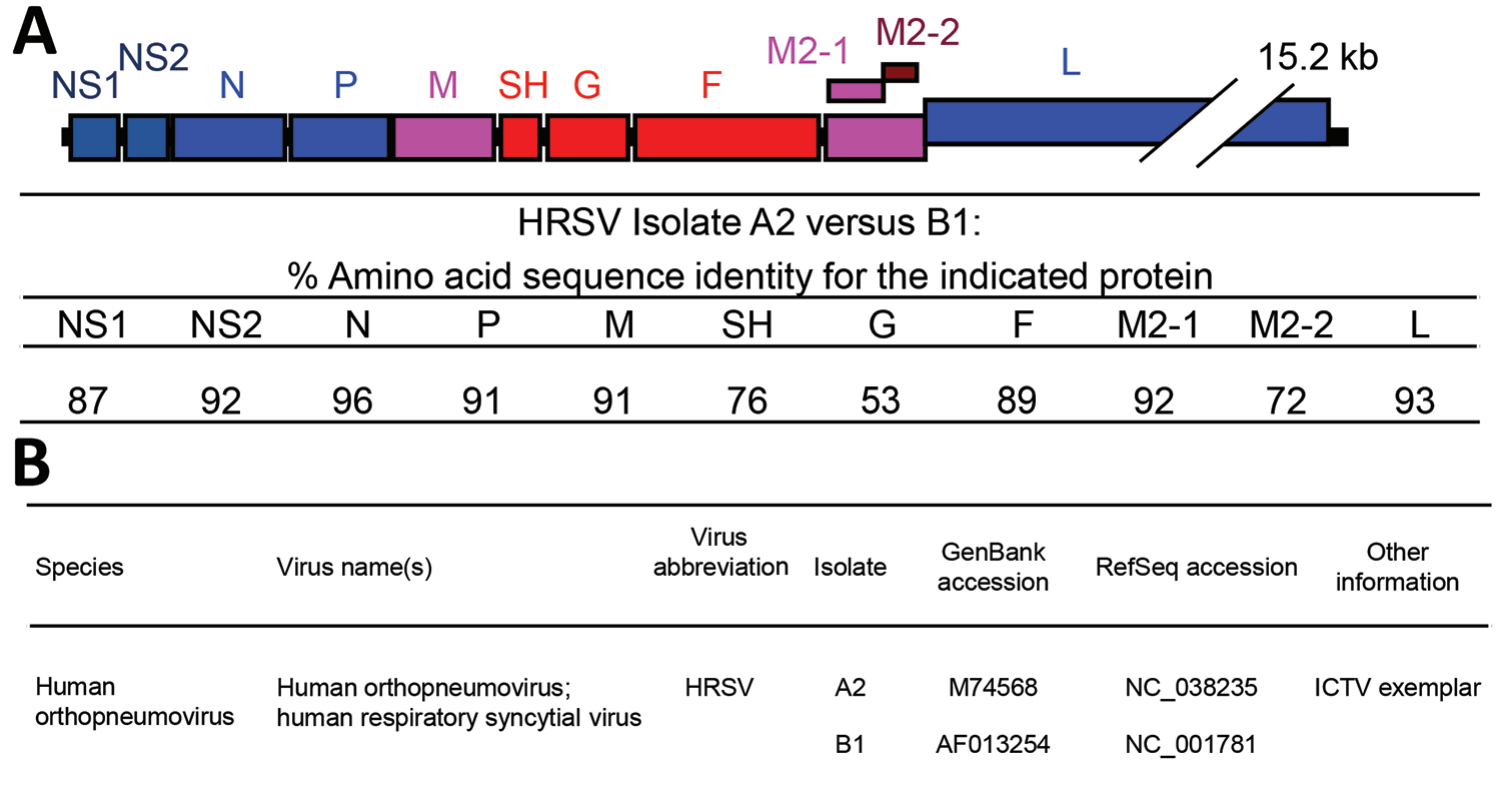
A) Schematic overview of the HRSV gene order and comparison of the amino acid identities of the reference strains of subgroups A (HRSV A2, GenBank accession no. M74568/NC_038235) and B (HRSV B1, GenBank accession no. AF013254/NC_001781). B) ICTV-proposed species designation, virus name, and associated GenBank reference sequences. HRSV, human respiratory syncytial virus; ICTV, International Committee on Virus Taxonomy; RefSeq, reference sequence.

HRSV has a single-stranded nonsegmented negative-sense RNA genome ≈15,191–15,277 nt long (Figure 2, panel A) (*7*). The HRSV genome contains 10 genes, each encoding a separate mRNA with a single open reading frame (ORF) (Figure 2, panel A; Table), except for the M2 mRNA, which contains 2 overlapping ORFs. The 11 HRSV proteins are 2 nonstructural proteins (NS1 and NS2), nucleoprotein (N), phosphoprotein (P), matrix protein (M), small hydrophobic envelope protein (SH), attachment glycoprotein (G), fusion glycoprotein (F), the transcription processivity factor (M2–1), RNA regulatory factor (M2–2), and large RNA polymerase protein (L) (Table) (*24**,**25*). The F glycoprotein is the major viral neutralization and protective antigen, followed by the G glycoprotein (*26*).

**Table:** Widely accepted nomenclature for HRSV genes and proteins and gene annotation of strains HRSV A2 and HRSV B1*

Gene or genome region				Proteins			Open reading frame genome position, nt	
Annotation†	Genome position, nt†							
	Strain A2‡	Strain B1¶		Annotation	Abbreviation		Strain A2	Strain B1
Leader region	1–44	1–44						
NS1	45–576	45–577		Nonstructural protein 1	NS1		99–518	99–518
NS2	596–1098	594–1098		Nonstructural protein 2	NS2		628–1002	626–1000
N	1126–2328	1125–2327		Nucleoprotein	N		1141–2316	1140–2315
P	2330–3243	2331–3244		Phosphoprotein	P		2347–3072	2348–3073
M	3253–4210	3254–4208		Matrix protein	M		3262–4032	3263–4033
SH	4220–4629	4218–4630		Small hydrophobic protein	SH		4304–4498	4303–4500
G	4674–5596	4675–5600		Attachment glycoprotein	G		4689–5585	4690–5589
F	5649–7551	5653–7552		Fusion glycoprotein	F		5662–7386	7666–7390
M2	7598–8558	7609–8568		Matrix protein 2				
				Matrix protein M2–1	M2–1		7607–8191	7618–8205
				Matrix protein M2–2	M2–2		8160–8432	8171–8443
L	8491–15068	8501–15080		Polymerase protein	L		8499–14996	8509–15009
Trailer region	15069–15223	15081–15225						

*HRSV, human respiratory syncytial virus. †Nucleotide annotations for leader and trailer region, and for indicated HRSV genes from the first nucleotide of the gene start signal [GGGGCAAAT(A/G); GGAGCAAAAT in case of the L gene] through the last adenosine residue of the gene end signal [AGT(T/A)A(T/A/G)(A/T)(A/T)(A/T)A_n_]. ‡HRSV/A/USA/A2/2015 (*45*); GenBank accession number KT992094. ¶HRSV/B/USA/B1/1985/B1 (*46*); GenBank accession number AF013254.1.

## HRSV Subgroups and Genotype Designations: Status and Outlook

HRSV subgroups A and B exhibit genomewide nucleotide and amino acid divergence (Figure 2, panel A) (*25**,**27*). The reference sequences for the 2 subgroups are derived from strains HRSV A2 (*28**–**31*; GenBank accession number M74568.1; RefSeq accession number NC_038235) and HRSV B1 (*32*; GenBank accession number AF013254.1; RefSeq accession number NC_001781.1; Figure 2, panel B). F glycoprotein sequences between the 2 subgroups are well conserved (89% aa identity), whereas the G glycoproteins are the most divergent (53% aa identity between the subgroups) among the HRSV proteins (Figure 2, panel A) and undergo continuous molecular evolution. The ectodomain of the G glycoproteins of both subgroups contains a conserved central domain, representing an important antigenic site, flanked by 2 hypervariable domains (*33*). Except for the central conserved region, the antigenic cross-reactivity between G glycoproteins of the 2 subgroups is low (*26*).

Because the G ORF exhibits the greatest degree of genetic variability between isolates, it is most commonly used for studies on the molecular evolution of HRSV. The genetic variability of HRSV strains over time has been commonly determined by sequencing the distal C-terminal third of the G ORF, which includes the second hypervariable domain. The variability in the G ORF is characterized by a high rate of nonsynonymous nucleotide changes, suggesting that evolution may be driven by immune pressure, even though this factor may be partially antibody independent (*34*). It is likely that variability in the G protein contributes to the capacity of HRSV to cause yearly outbreaks in the community (*35**–**37*). The nomenclature proposal outlined herein will be useful for the sequence analyses required to follow the molecular evolution of HRSV.

In a parallel effort, several research groups are working together on a genotyping proposal to provide a consensus on uniform genotype designations (*38*,*39*). As virus evolution continues, we expect new genotypes to emerge and older genotypes to become extinct. HRSV genotyping designations will need to capture present molecular evolutionary status and be adaptable to changes and will need to be reevaluated periodically by a global consortium.

## Nomenclature Proposal for HRSV Strains and Isolates

For molecular epidemiology studies, a concise standard for short identifiers of specific HRSV sequences, suitable for the short definition lines that give context to a sample and its derived sequence, would be useful. Ideally, concise standardized identifiers should convey key information about each individual sequence in an alignment or phylogram, including source, date, and type, if known. Here, we aim to define this type of common naming convention for HRSV samples and isolates. We also propose using standard names and appropriate annotations for HRSV genes, provide examples to guide the annotation of sequence data during the sequence submission process, and suggest how to submit metadata associated with the source materials of HRSV sequences.

## Definition Lines for HRSV Sequence Submissions

**Figure 3:**
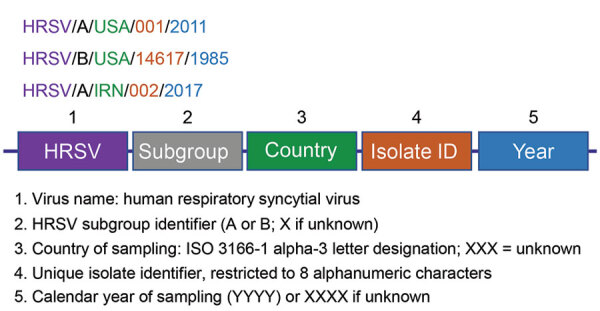
Schematic representation of the 5 consensus nomenclature elements of HRSV strains and isolates, with examples (top) and an explanation of each element (bottom). HRSV, human respiratory syncytial virus; ID, identification number; ISO, International Organization for Standardization.

GenBank records available through the National Center for Biotechnology Information (NCBI) are identified by 2 elements: a unique alphanumeric accession number and a definition line. The definition line is the portion of the identifier commonly associated with GenBank records shown in BLAST results and other searches. Definition lines are generated by the submitter during the sequence submission process and include the species and isolate name (e.g., *Human orthopneumovirus* isolate HRSV/A/USA/001/2011, complete genome [proposed]). (*40*). We propose a standardized format to capture 5 sample-specific parameters of HRSV-positive clinical samples or isolates to be included in sequence definition lines compatible with database naming requirements (Figure 3), in this specific order: [virus name abbreviation]/[HRSV subgroup]/[geographic identifier]/[unique sequence identifier]/[year of sampling].

### Elements of Sequence Definition

#### I. Organism name; virus name abbreviation: HRSV

ICTV’s species name, *Human orthopneumovirus* (*7*), will be reflected as HRSV in the NCBI definition line. During submission to databases, the organism name can be entered as either Human orthopneumovirus or human respiratory syncytial virus. The abbreviation HRSV should be used in the definition line regardless of which organism name is provided.

#### II. HRSV subgroup: A or B; X, if unknown

#### III. Geographic identifier for the location of sampling. Because individual HRSV research networks have predefined requirements, we suggest some flexibility for this field:

- If not specified by a research network, the ISO 3166–1 α-3 letter country code (https://www.iso.org/iso-3166-country-codes.html) should be used to indicate the country of sampling (XXX, if unknown). We strongly suggest that submitters provide any more specific geographic information on the sampling location (e.g., city or state) in metadata fields rather than in the definition line.
- The WHO Global RSV Surveillance Project plans to use just the simple English-language name for the country.
- Individual national studies may require a state, province, or city designation, in addition to the mandatory country name. If required, a period should be used to set off the country name ([state/province/city].[country name]).

#### IV. Unique isolate identifier

This field must be restricted to 8 alphanumeric characters. Underscores are permitted, but neither other special characters (e.g., /, %, $, @) nor spaces can be used. Controversial names or phrases, names of prominent people, and trademarked names or phrases cannot be used. To prevent duplication of sample or isolate identifiers by different groups, we recommend inserting a lettercode identifying a study or institute before the sample number. For example, unique isolate identifiers for samples from the INFORM-RSV study might use “INF” followed by a number (e.g., INF001 in HRSV/A/COUNTRY/INF001/2019).

#### V. Year of sampling; YYYY or XXXX, if unknown.

### Examples of Sequence Definition Lines Using Proposed Nomenclature

- HRSV/A/USA/001/2011
- HRSV/B/Denver.USA/14617/1985
- HRSV/A/IRN/001/2017
- HRSV/A/Iran/001/2017
- HRSV/X/IRN/001/2017 (subgroup unknown)
- HRSV/B/New_Zealand/FR123/2020

Our nomenclature proposal prioritizes a short, concise definition line that will be easy to use in the laboratory, easily readable, and be a uniform system for HRSV in public databases. Additional host, virus, location or temporal information if desired could be submitted in metadata fields, which would allow researchers, epidemiologists, and database users to apply specific metadata filters, as needed for data retrieval and specific applications, analyses, or for displaying designations, such as in dendrograms.

## Terminology for Annotations

**Figure 4:**
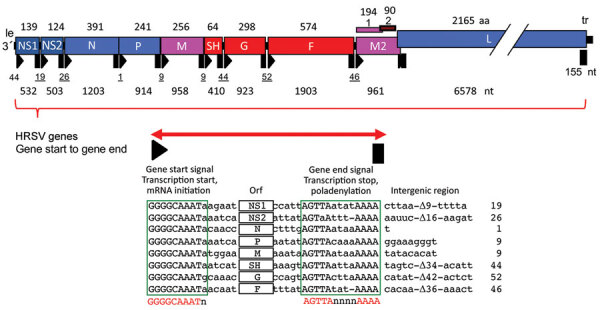
Schematic aid to gene annotations of HRSV whole-genome sequences. An HRSV gene comprises the sequence from the first nucleotide of the conserved HRSV gene start signal (GGGGCAAATa) to the last adenosine residue of the HRSV gene end signal (AGTTAnnnnAAAA) (*25*,*41*). Gene start signals are represented by black triangles, and gene end signals are shown as black rectangles, separated by intergenic regions (underlined). Note the M2/L gene overlap (annotations derived from HRSV A2; GenBank accession no. M74568/NC_038235). le, leader region; HRSV, human respiratory syncytial virus; tr, trailer region.

To support efficient data analysis, uniform designations must be used at the database submission stage. Commonly accepted names for HRSV genes and proteins are shown in the table. An HRSV gene comprises a gene start signal GGGGCAAAT(A/G), an ORF with adjacent noncoding regions, if present, and the gene end signal through the last adenosine residue [AGT(T/A)A(T/A/G)(A/T)(A/T)(A/T)A_n_] (Figure 4; *41*). Each HRSV gene contains a single ORF, except for the M2 gene, which has 2 overlapping ORFs, M2–1 and M2–2. Nucleotide annotations of genes and ORFs for the HRSV A2 (Figure 4) and HRSV B1 reference sequences are shown in the table.

## Metadata for HRSV Sequence Submissions

What is the most pertinent host data will depend on the interests and objectives of individual study groups. For example, when studying HRSV in a pediatric setting, prematurity may be of interest, but when studying HRSV in an adult setting, researchers may be more interested in whether participants are immunocompromised. We suggest information that could be included in metadata fields for HRSV:

### 1. Isolation source: sample type (upper or lower airways)

Viral RNA can be extracted directly from a clinical sample, from an isolate grown in cell culture, or possibly from a cDNA-derived recombinant virus. The sources of sequences from isolated viruses can be identified by the following designations:
  - wt: wild-type; sequences derived from RNA extracted directly from clinical specimens
  - tc: tissue culture; sequences derived from RNA extracted from HRSV isolates propagated in tissue culture
  - rec: recombinant; sequences of cDNA-derived recombinant virus (including vaccine strain)

### 2. Host: 

#### *Homo sapiens*; subject age. Indicate years and months if <5 years of age, years only if ≥5 years of age. Sex should be spelled out if known.

### 3. Country, state, and (nearest) city of sampling. 

#### Metadata information must include the full country name (not the 3-letter abbreviation) from the NCBI list of accepted country designations (https://www.ncbi.nlm.nih.gov/GenBank/collab/country). City, state, or province can also be included. Names should be written based on the standard ASCII letters including spaces if required (https://www.nist.gov/system/files/documents/2021/03/23/ansi-nist_2010_traditional_encoding.pdf). Geolocation coordinates of the location where sampling took place should be included if known.

### 4. Collection date

####  We highly recommend that the exact date of specimen collection (DD-Mon-YYYY format; e.g., 17-Feb-2002) be used; if exact date is not known, at least the month and year should be indicated (Mon-YYYY format).

### 5. Genotype according to the consensus in genotype classification by an HRSV working group [in progress (*38*,*39*)]. 

#### Associated with the International RSV Society, a special interest group of the International Society for Influenza and other respiratory viruses https://www.isirv.org/site/index.php/special-interest-groups/international-respiratory-syncytial-virus-society.

### 6. Metadata on the patient-host and the clinical disease should be included in the notes field in a structured format. Protected personally identifiable health information will be excluded from metadata submissions.

- If >6 months of age, birthweight and gestational age at birth.
- Significant pediatric co-morbidities, including prematurity, congenital cardiac disease, and broncho-pulmonary dysplasia (BPD).
- Twin? (Y/N)
- Exposed to specific HRSV therapeutic, vaccine, antibody, or antiviral? (Y/N)
- Viral or bacterial co-infections, if known; pathogen species should be spelled out.
- Adult underlying conditions, such as chronic obstructive pulmonary disease (COPD) or asthma, or altered immune status (e.g., immunocompromised, bone marrow transplant recipient).
- Disease outcomes. Five grades are distinguished:
  - No medical care.
  - Outpatient or emergency room.
  - Hospital admission.
  - ICU admission.
  - Death.

For NCBI submissions, data can be entered through the web interface, or uploaded as tab-delimited text files. Sequences can be uploaded in FASTA format (https://blast.ncbi.nlm.nih.gov; Appendix), with associated metadata provided in a plain text, tab-delimited, source modifier table (Appendix Table 1) and gene or protein annotations provided in a plain text, tab-delimited, 5-column feature table (Appendix Table 2).

## Outlook

Molecular surveillance has revealed that multiple HRSV genotypes circulate simultaneously in communities. Circulating genotypes often vary between communities, and circulation patterns within a community can change from year to year. Extended monitoring of circulating viruses is necessary to better understand transmission and molecular evolution (*42*). As HRSV vaccine candidates and antivirals are being developed, molecular epidemiology studies may reveal potential effects of prevention strategies on viral evolution and possible antibody-escape variants. Timely sharing of HRSV data worldwide through the use of public databases is essential. We propose that sequence data be uploaded to publicly accessible databases, such as NCBI (*31*). Although NCBI is the most complete repository for HRSV sequence information, studies may require that sequences first be submitted to other databases, such as GISAID (https://www.gisaid.org).

Public access will provide useful availability for investigators to submit, query, and analyze HRSV sequence data, enabling the evolutionary analysis of sequence diversity within or between HRSV genotypes. The utility of public access has been clearly demonstrated with the emergence of severe acute respiratory syndrome coronavirus 2 (SARS-CoV-2) and the critical role that genetic sequence analysis has played. Notably, a nomenclature model for SARS-CoV-2 similar to what we have proposed for HRSV has been adopted, although some differences remain between databases (e.g., NCBI, SARS-CoV-2/human/USA/COVID20–1096/2020; GISAID, hCoV-19/Australia/VIC12/2020). When an HRSV vaccine becomes available, high-quality, geographically representative, country-specific data on circulating strains and rich datasets of well-curated, standardized, and parsable data will be required to monitor and trace possible evolutionary changes in response to vaccine-induced selective pressure (*43**,**44*). This proposal will profit from strong support by members of the International RSV Society, a special interest group of the International Society for Influenza and other Respiratory Virus Diseases (https://isirv.org), members of the WHO Global RSV Surveillance Project, and the HRSV research community.

Appendix Background genomic sequencing information on human respiratory syncytial virus.
