# Supplementary material for: Proposal for Human Respiratory Syncytial Virus Nomenclature below the Species Level
Source: Emerg Infect Dis. 2021 Jun;27(6):e204608. doi: 10.3201/eid2706.204608 (PMC8153853; doi:10.3201/eid2706.204608)
Supplement: Appendix — Background genomic sequencing information on human respiratory syncytial virus. [file 20-4608-Techapp-s1.pdf]

Article DOI: https://doi.org/10.3201/eid2706.204608

Proposal for Human Respiratory Syncytial Virus Nomenclature below the Species Level

Appendix

Appendix Table 1. Virtual examples of typical sequence-associated data, not based on existing primary data*

Seq	Isolate	Source	Sex/ age	Lab host	Location	Date	Geno	Notes	Sub
rsv 011	HRSV/A/PHL/pim16223/ 2016	Wt; sputum	M/ 3 mo	NA	Philippines	Dec 2016	ON1	Wt/gest age at birth: 7 lb/ 38 w; twin: no; HRSV therapy: no; co-infection: Strep A; severity: ER	A
rsv 012	HRSV/B/ARG/352162/ 2019	Wt; nasopharyngeal aspirate	F/ 2 y, 7 mo	NA	Buenos Aires, Argentina	May 2019	TBD	Co-morbidities: broncho-pulmonary dysplasia; severity: hospital admission	B
rsv 013	HRSV/A/Piura.PER/ PIU048/2020	TC; nasal swab	F/ 87 y	HEp-2 cells x2	Piura, Peru	Apr 2020	TBD	Co-infection: influenza A; severity: no medical care	A

*Geno, genotype; lb, pound; Seq, sequence identification; Sub, subtype; TBD, to be determined; TC, tissue culture; wt, weight

Appendix Table 2. Virtual examples of typical sequence-associated data, not based on existing primary data*

Start location (first nt)	Stop location (last nt)	Feature name	Qualifier (product or gene)	Qualifier value (designation)
>Feature rsv011				
4	576	Gene		
			Gene	NS1
99	518	CDS		
			Product	Nonstructural protein 1
596	1098	Gene		
			Gene	NS2
628	1002	CDS		
			Product	Nonstructural protein 2
112	2326	Gene		
			Gene	N
1140	2315	CDS		
			Product	Nucleoprotein
2330	3242	Gene		
			Gene	P
2347	3072	CDS		
			Product	Phosphoprotein
3246	4203	Gene		
			Gene	M
3255	4025	CDS		
			Product	Matrix protein
4211	4621	Gene		
			Gene	SH
4295	4489	CDS		
			Product	Small hydrophobic protein
4666	5659	Gene		
			Gene	G
4681	5646	CDS		
			Product	Attachment glycoprotein
5713	7615	Gene		
			Gene	F
5726	7450	CDS		
			Product	Fusion glycoprotein
7660	8620	Gene		

Start location (first nt)	Stop location (last nt)	Feature name	Qualifier (product or gene)	Qualifier value (designation)
			Gene	M2
7669	8253	CDS		
			Product	M2-1 protein
8222	8494	CDS		
			Product	M2-2 protein
8552	15124	Gene		
			Gene	L
8561	15058	CDS		
			Product	Polymerase protein
>Feature rsv012				
4	576	Gene		
			Gene	NS1
99	518	CDS		
			Product	Nonstructural protein 1
596	1098	Gene		
			Gene	NS2
628	1002	CDS		
			Product	Nonstructural protein 2
112	2326	Gene		
			Gene	N
1140	2315	CDS		
			Product	Nucleoprotein
2330	3242	Gene		
			Gene	P
2347	3072	CDS		
			Product	Phosphoprotein
3246	4203	Gene		
			Gene	M
3255	4025	CDS		
			Product	Matrix protein
4211	4621	Gene		
			Gene	SH
4295	4489	CDS		
			Product	Small hydrophobic protein
4666	5659	Gene		
			Gene	G
4681	5646	CDS		
			Product	Attachment glycoprotein
5713	7615	Gene		
			Gene	F
5726	7450	CDS		
			Product	Fusion glycoprotein
7660	8620	Gene		
			Gene	M2
7669	8253	CDS		
			Product	M2-1 protein
8222	8494	CDS		
			Product	M2-2 protein
8552	15124	Gene		
			Gene	L
8560	15058	CDS		
			Product	Polymerase protein
>Feature rsv013				
<1	333	Gene		
			Gene	NS1
<1	275	CDS		
			Product	Nonstructural protein 1
353	855	Gene		
			Gene	NS2
385	759	CDS		
			Product	Nonstructural protein 2
882	2085	Gene		
			Gene	N
897	2072	CDS		
			Product	Nucleoprotein
2095	3007	Gene		
			Gene	P

Start location (first nt)	Stop location (last nt)	Feature name	Qualifier (product or gene)	Qualifier value (designation)
2112	2837	CDS		
			Product	Phosphoprotein
3011	3968	Gene		
			Gene	M
3020	3790	CDS		
			Product	Matrix protein
3976	4385	Gene		
			Gene	SH
4060	4254	CDS		
			Product	Small hydrophobic protein
4430	5351	Gene		
			Gene	G
4445	5338	CDS		
			Product	Attachment glycoprotein
5405	7307	Gene		
			Gene	F
5418	7142	CDS		
			Product	Fusion glycoprotein
7352	8312	Gene		
			Gene	M2
7361	7945	CDS		
			Product	M2-1 protein
7914	8186	CDS		
			Product	M2-2 protein
8245	>14615	Gene		
			Gene	L
8253	>14615	CDS		
			Product	Polymerase protein

*CDS, coding sequence (coding region of gene)

Human respiratory syncytial virus genome

```
>rsv011
ACGCGAAAAAATGCGTACAACAAACTTGCGTAAACCAAAAAAATGGGGCAAATAAGAATTTGATAAGTACCACTTAAATT
TAACTCCTTTGGTTAGAGATGGGCAGCAACTCATTGAGTATGATAAAAGTTAGATTGCAAAATCTGTTTGACAATGATGA
AGTAGCATTGTTAAAAATAACATGCTATACTGACAAATTAATACAGTTAACTAATGCTTTGGCTAAGGCAGTTATACATA
CAATCAAATTGAATGGCATTGTATTTGTGCATGTTATTACAAGTAGTGATATTTGCCCTAATAATAATATTGTAGTGAAA
TCCAATTTCACAACAATGCCAGTATTACAAAATGGAGGTTATATATGGGAAATGATGGAATTAACACACTGCTCTCAACC
TAATGGCCTAATAGATGACAATTGTGAAATTAAATTCTCCAAAAAACTAAGTGATTCAACAATGACCAATTATATGAATC
AATTATCTGAATTACTTGGATTTGACCTCAATCCATAAATCATAATAAATATCAACTAGCAAATCAATGTCACTAACACC
ATTAGTTAATATAAAACTTGACAGAAGATAAAAATGGGGCAAATAAATCAATTCAGCCGACCCAACCATGGACACAACAC
ACAATGATACCACACCACAAAGACTGATGATCACAGACATGAGACCATTATCGCTTGAGACTATAATAACATCTCTAACC
AGAGATATCATAACACATAAATTTATATACTTGATAAATCATGAATGCATAGTGAGAAAACTTGATGAAAGACAGGCCAC
ATTTACATTTCTGGTCAACTATGAAATGAAACTATTGCACAAAGTGGGAAGCACTAAATATAAAAAATATACTGAATACA
ACACAAAATATGGCACTTTCCCTATGCCAATATTTATCAATCATGATGGGTTCTTAGAATGCATTGGCATTAAGCCTACC
AAGCACACACCCATAATATACAAGTATGATCTCAATCCATGAATATCAAACCAAGATTCAAACAATCCGAAATAACAACT
TTATGCATAATCACACTCCATAGTCCAAATGGAGCCTGAAAATTATAGTTATTTAAAATTAAGGAGAGACATAAGATGAA
AGATGGGGCAAATACAAAAATGGCTCTTAGCAAAGTCAAGTTGAATGATACACTCAACAAAGATCAACTTCTATCATCCA
GCAAATATACCATCCAACGGAGCACAGGAGACAGCATTGACACTCCTAATTATGATGTGCAGAAACACATTAATAAGTTA
TGTGGCATGTTATTAATCACAGAAGATGCTAATCATAAATTCACTGGGTTAATAGGTATGTTATATGCTATGTCTAGATT
AGGAAGAGAAGACACCATAAAAATACTTAAAGATGCGGGATATCATGTTAAGGCAAATGGAGTGGATGTAACAACACATC
GTCAAGACATTAATGGGAAAGAAATGAAATTTGAAGTGTTAACATTAGCAAGCTTAACAACTGAAATTCAAATCAACATT
GAGATAGAATCTAGAAAATCCTACAAAAAAATGCTAAAAGAAATGGGAGAGGTGGCTCCAGAATACAGGCATGACTCTCC
AGATTGTGGGATGATAATATTATGTATAGCAGCATTAGTAATAACCAAATTAGCAGCAGGAGATAGATCAGGTCTTACAG
CTGTGATTAGGAGAGCTAATAATGTCCTAAAAAATGAAATGAAACGTTACAAAGGTTTATTACCCAAGGATATAGCCAAC
AGCTTCTATGAAGTGTTTGAAAAATATCCTCACTTTATAGATGTTTTTGTTCATTTTGGTATAGCACAATCTTCTACCAG
AGGTGGCAGTAGAGTTGAAGGGATTTTTGCAGGATTGTTTATGAATGCCTATGGTGCAGGGCAAGTGATGTTACGGTGGG
GGGTCTTAGCAAAATCAGTTAAAAACATTATGTTAGGACACGCTAGTGTACAAGCAGAAATGGAACAAGTTGTGGAGGTG
```

TATGAGTATGCTCAGAAATTGGGTGGAGAAGCAGGATTCTACCATATATTGAACAACCCAAAAGCATCACTATTATCTTT
GACTCAATTTCCTCACTTCTCTAGTGTAGTATTGGGCAATGCTGCTGGCCTAGGCATAATGGGAGAATACAGAGGTACAC
CAAGGAATCAAGATTTATATGATGCTGCAAAAGCATATGCTGAACAACTCAAAGAAAATGGTGTGATTAACTACAGTGTA
TTAGATTTGACAGCAGAAGAACTAGAGGCTATCAAACATCAGCTTAATCCAAAAGATAATGATGTAGAGCTTTGAGTTAA
TAAAAAGGTGGGGCAAATAAATCATCATGGAAAAGTTTGCTCCTGAATTCCATGGAGAAGATGCAAACAACAGAGCCACC
AAATTCCTAGAATCAATAAAGGGCAAATTCACATCACCCAAAGATCCCAAGAAAAAAGATAGTATCATATCTGTCAACTC
AATAGATATAGAAGTAACCAAAGAAAGCCCTATAACATCAAATTCAACCATTATAAACCCAATAAATGAGACAGATGATA
CTGTAGGGAACAAGCCCAATTATCAAAGAAAGCCTCTAGTAAGTTTCAAAGAAGACCCTATGCCAAGTGATAATCCTTTT
TCAAAACTATACAAAGAAACCATAGAAACATTTGATAACAATGAAGAAGAATCTAGCTATTCATATGAAGAAATAAATGA
TCAGACAAACGATAATATAACAGCAAGATTAGATAGGATTGATGAGAAATTAAGTGAAATACTAGGAATGCTTCACACAT
TAGTAGTAGCGAGTGCAGGACCCACATCTGCTCGGGATGGTATAAGAGATGCCATGGTTGGTTTAAGAGAAGAAATGATA
GAAAAAATCAGAACTGAAGCATTAATGACCAATGACAGACTAGAAGCTATGGCAAGACTCAGGAATGAAGAAAGTGAAAA
GATGGCAAAAGACACATCAGATGAAGTGTCTCTCAATCCAACATCAGAGAAACTGAACAACCTGTTGGAAGGGAATGATA
GTGACAATGATCTATCACTTGAAGATTTCTGATTAGCTACCAAACTGTACATCAAAACACAACACCAATAGAAAACCAAC
AAACAAACCAACTCACCCATCCAACCAAACATCTATCTGCTGATTAGCCAACCAGCCAAAAAACAACCAGCCAATCTAAA
ACTAGCCACCCGGAAAAAATCGATACTATAGTTACAAAAAAAGATGGGGCAAATATGGAAACATACGTGAATAAACTTCA
CGAGGGCTCCACATACACAGCTGCTGTTCAATACAATGTCCTAGAAAAAGACGATGATCCTGCATCACTTACAATATGGG
TGCCCATGTTCCAATCATCCATGCCAGCAGATCTACTCATAAAAGAACTAGCCAATGTCAATATACTAGTGAAACAAATA
TCCACACCCAAGGGACCCTCATTAAGAGTCATGATAAACTCAAGAAGTGCAGTGCTAGCACAAATGCCCAGCAAATTTAC
CATATGTGCCAATGTGTCCTTGGATGAAAGAAGCAAGCTAGCATATGATGTAACCACACCCTGTGAAATTAAGGCATGCA
GTCTAACATGCCTAAAATCAAAAAATATGTTAACTACAGTTAAAGATCTCACTATGAAAACACTCAACCCAACACATGAC
ATCATTGCTTTATGTGAATTTGAAAATATAGTAACATCAAAAAAAGTCATAATACCAACATACCTAAGATCTATCAGCGT
CAGAAATAAAGATCTGAACACACTTGAAAATATAACAACCACTGAATTCAAAAATGCCATTACAAATGCAAAAATCATCC
CTTACTCAGGATTACTGTTAGTCATCACAGTGACTGACAACAAAGGAGCATTCAAATACATAAAGCCACAAAGTCAATTC
ATAGTAGATCTTGGAGCCTACCTAGAAAAAGAAAGTATATATTATGTTACAACAAATTGGAAGCACACAGCTACACGATT
TGCAATCAAACCCATGGAAGATTAACCTTTTTCCTATACATCAATGAGTAGATTCATACAAACTTTCTAACTACATTCTT
CACTTCACAATCATAATCACCAACCCTCTGTGGTTCAATCAATCAAACAAAACTCATCAGGAGTTCCAGATCATCCCAAG
TCATTGTTCATCAGATCCAGTACTCAAATAAGTTAATAAAAAATCCACATGGGGCAAATAATCATTGAGGGAAATCCAAC
TAATCACAACATCTGTCAACATAGACAAGTCAACACGCTAGATAAAATCAACCAATGGAAAATACATCCATAACTATAGA
ATTCTCAAGCAAATTCTGGCCTTACTTTACACTAATACACATGATAACAACAATAATCTCTTTGATAATCATAATCTCCA
TCATGATTGCAATACTAAACAAACTCTGCGAATATAATGTATTCCATAACAAAACCTTTGAGCTACCAAGAGCTCGAGTC
AATACATAGCATTCACCAATCTGATAGCTCAAAACAGTAACCTTGCATTTGTAAATGAACTACCCTCACTTCTTCACAAA
ACCACATCAACATCTCACCATGCAAGCCATCATCTATACCATAAAGTAGTTAATTAAAAAATAGTCATAACAATGAACTA
GGATATTAAGACCAAAAACAACGCTGGGGCAAATGCAAACATGTCCAAAACCAAGGACCAACGCACCGCCAAGACACTAG
AAAGGACCTGGGACACTCTCAATCATCTGTTATTCATATCATCGTGCTTATACAAGTTAAATCTTAAATCTATAGCACAA
ATCACATTATCTATTTTGGCAATGATAATCTCAACCTCACTTATAATTGCAGCCATCATATTCATAGCCTCGGCAAACCA
CAAAGTCACACTAACAACTGCAATCATACAAGATGCAACGAACCAGATCAAGAACACAACCCCAACATACCTCACCCAGA
ATCCCCAGCTTGGAATCAGCTTCTCCAATCTGTCCGGAACTACATCACAATCCACCACCATACTAGCTTCAACAACACCA
AGTGCTGAGTCAACCCCACAATCCACAACAGTCAAGATCAAAAACACAACAACAACCCAAATATTACCTAGCAAACCCAC
CACAAAACAACGCCCAAATAAACCACAAAACAAACCCAACAATGATTTTCACTTTGAAGTGTTCAATTTTGTACCCTGCA
GCATATGCAGCAACAATCCAACCTGCTGGGCCATCTGCAAGAGAATACCAAACAAAAAACCTGGAAAGAAAACCACCACC
AAGCCCACAAAAAAACCAACCCTCAAGACAACCAAAAAAGATCCCAAACCTCAAACCACAAAACCAAAGGAAGTACTCAC
TACCAAGCCTACAGGAAAGCCAACCATCAACACCACCAAAACAAACATCAGAACTACACTGCTCACCTCCAACACCAAAG
GAAATCCAGAACACACAAGTCAAGAGGAAACCCTCCACTCAACCACCTCCGAAGGCTATCTAAGCCCATCACAAGTCTAT
ACAACATCCGGTCAAGAGGAAACCCTCCACTCAACCACCTCCGAAGGCTATCTAAGCCCATCACAAGTCTATACAACATC
CGAGTACCTATCACAATCTCTATCTTCATCCAACACAACAAAATGATAGTCATTAAAAAGCGTATTGTTGCAAAAAGCCA
TGACCAAATCAAACAGAATCAAAATCAACTCTGGGGCAAATAACAATGGAGTTGCCAATCCTCAAAACAAATGCTATTAC
CACAATCCTTGCTGCAGTCACACTCTGTTTCGCTTCCAGTCAAAACATCACTGAAGAATTTTATCAATCAACATGCAGTG
CAGTTAGCAAAGGCTATCTTAGTGCTCTAAGAACTGGTTGGTATACTAGTGTTATAACTATAGAATTAAGTAATATCAAG
GAAAATAAGTGTAATGGTACAGACGCTAAGGTAAAATTAATAAAACAAGAATTAGATAAATATAAAAATGCTGTAACAGA
ATTGCAGTTGCTCATGCAAAGCACACCAGCAGCCAACAGTCGAGCCAGAAGAGAACTACCAAGATTTATGAATTATACAC
TCAACAATACCAAAAACACCAATGTAACATTAAGTAAGAAAAGGAAAAGAAGATTTCTTGGATTTTTGTTAGGTGTTGGA
TCTGCAATCGCCAGTGGCATTGCCGTATCCAAGGTCCTGCACCTAGAAGGGGAAGTGAACAAAATCAAAAGTGCTCTACT
ATCCACAAACAAGGCTGTAGTCAGCTTATCTAATGGAGTCAGTGTCTTAACCAGCAAGGTGTTAGACCTCAAAAACTATA
TAGATAAACAGTTGTTACCTATTGTTAACAAGCAAAGCTGCAGCATATCAAACATTGAAACTGTGATAGAGTTCCAACAA

AAGAACAACAGACTACTAGAGATTACCAGAGAATTTAGTGTTAATGCAGGTGTAACTACACCTGTAAGCACTTATATGTT
AACTAATAGTGAGTTATTATCATTAATCAATGATATGCCTATAACAAATGATCAGAAAAAGTTAATGTCCAGCAATGTTC
AAATAGTTAGACAGCAAAGTTACTCTATCATGTCAATAATAAAAGAGGAAGTCTTAGCATATGTAGTACAATTACCACTA
TATGGTGTAATAGATACTCCTTGTTGGAAACTACACACATCCCCTCTATGTACAACCAACACAAAGGAAGGATCCAACAT
CTGCTTAACAAGAACCGACAGAGGATGGTACTGTGACAATGCAGGATCAGTATCCTTTTTCCCACAAGCTGAAACATGTA
AAGTTCAATCGAATCGGGTGTTTTGTGACACAATGAACAGTTTAACATTACCAAGTGAGGTAAATCTCTGCAACATTGAC
ATATTCAACCCCAAATATGATTGCAAAATTATGACTTCAAAAACAGATGTAAGCAGCTCCGTTATCACATCTCTAGGAGC
CATTGTGTCATGCTATGGCAAAACCAAATGTACAGCATCCAATAAAAATCGTGGGATCATAAAGACATTCTCTAACGGGT
GTGATTATGTATCAAATAAGGGGGTGGATACTGTGTCTGTAGGTAATACATTATATTATGTAAATAAGCAAGAAGGCAAA
AGTCTCTATGTAAAAGGTGAACCAATAATAAATTTCTATGATCCATTAGTGTTCCCCTCTGATGAATTTGATGCATCAAT
ATCTCAAGTCAATGAGAAAATTAATCAGAGTCTAGCATTTATCCGTAAATCAGATGAATTATTACATAATGTAAATGCTG
GTAAATCCACCACAAATATCATGATAACTACCATAATTATAGTAATTATAGTAATATTGTTAGCATTAATTGCAGTTGGA
CTGCTTCTATACTGCAAGGCCAGAAGCACACCAGTCACATTAAGTAAGGATCAACTGAGTGGTATAAATAATATTGCATT
TAGTAACTGAATAAAAATAGCACCTAATCATATTCTTACAATGGTTCGCTATTTGACCATAGATAACCCATCTATCATTA
GATTATCCTAAAATTTGAACTTCATCACAACTTTCATCTATAAACCATCTCACTTACACTTTTTAAGTGGATTCCTATTT
TATAGTTATATAAAACAATTGAATACCAAATTAACTTACTATTTGTAAAAATGAGAACTGGGGCAAATATGTCACGAAGG
AATCCTTGCAAATTCGAAATTCGAGGTCATTGCTTGAATGGTAAAAGGTGTCATTTTAGTCATAATTATTTTGAATGGCC
ACCCCATGCACTGCTTGTAAGACAAAACTTTATGTTAAACAGAATACTTAAGTCTATGGATAAAAGCATAGATACTTTGT
CAGAAATAAGTGGAGCTGCAGAGTTGGACAGAACAGAAGAGTATGCCCTCGGTGTAGTTGGAGTGCTAGAGAGTTATATA
GGATCAATAAATAATATAACTAAACAATCAGCATGTGTTGCCATGAGCAAACTCCTTACTGAACTCAACAGCGATGACAT
AAAAAAACTAAGGGACAATGAAGAGCCAAACTCACCCAAAGTAAGAGTGTACAATACTGTCATATCATATATTGAAAGCA
ACAGGAGGAACAATAAACAAACTATCCATCTGTTAAAAAGATTGCCAGCAGACGTATTGAAGAAAACCATCAAAAACACA
TTGGATATCCACAAGAGCATAACCATCAATAACCCAAAAGAATCAACTGTTAGTGATACGAACGACCATGCCAAAAATAA
TGATACTACCTGACAAATATCCTTGTAGTATAAATTCCATACTAATAACAAGTAATTGTAGAGTCACTATGTATAATCAA
AAAAACACACTATATATCAATCAAAACAACCAAAATAACCATATATACCCACCGGATCAACCATTCAATGAAATCCATTG
GACCTCTCAAGACTTGATTGATGCAACTCAAAATTTTCTACAACATCTAGGTATTACTGATGATATATACACAATATATA
TATTAGTGTCATAATACTCAATCCTAATACTTACCACATCATCAAATTATTAACTCAAACAATTCAAGCTATGGGACAAA
ATGGATCCCATTATTAGTGGAAATTCTGCTAATGTTTATCTAACTGATAGTTATTTAAAAGGTGTTATTTCTTTCTCAGA
ATGTAACGCTTTAGGAAGTTACATATTCAATGGTCCTTATCTCAAAAATGATTATACCAACTTAATTAGTAGACAAAATC
CATTAATAGAACACATAAATCTAAAGAAACTAAATATAACACAGTCCTTAATATCTAAGTATCATAAAGGTGAAATAAAA
ATAGAAGAACCTACTTACTTTCAGTCATTACTTATGACATACAAGAGTATGACCTCGTCAGAACAGACTACTACTACTAA
TTTACTTAAAAAGATAATAAGAAGAGCTATAGAAATCAGTGATGTCAAAGTCTATGCTATATTGAATAAACTGGGGCTCA
AAGAAAAAGACAAGATTAAATCCAATAATGGACAAGATGAAGACAACTCAGTCATTACTACCATAATCAAAGATGATATA
CTTTTAGCTGTCAAGGATAATCAATCTCATCTTAAAGCAGACAAAAATCAATCCACAAAACAAAAAGATACAATCAAAAC
AACACTTTTGAAGAAATTAATGTGTTCGATGCAACATCCTCCATCATGGTTAATACATTGGTTTAATTTATACACACAAT
TAAACAGCATATTAACACAATATCGATCTAGTGAGGTAAAAAACCATGGTTTTATATTGATAGATAATCATACTCTTAGT
GGATTCCAATTTATTTTGAATCAATATGGTTGTATAGTTTATCATAAGGAACTCAAAAGAATTACTGTGACAACTTATAA
TCAATTCTTGACATGGAAAGATATTAGCCTTAGTAGATTAAATGTTTGTTTGATTACATGGATTAGTAACTGTTTGAACA
CATTAAACAAAAGCTTAGGCTTAAGATGTGGATTCAATAATGTTATCTTGACACAATTATTCCTTTATGGAGATTGTATA
CTAAAACTATTCCACAATGAGGGGTTCTACATAATAAAAGAGGTAGAGGGATTTATTATGTCTCTAATTTTAAATATAAC
AGAAGAAGATCAATTCAGAAAACGGTTTTATAATAGTATGCTCAACAACATCACAGATGCCGCCAACAAAGCTCAAAAAA
ATCTGCTATCAAGAGTATGTCATACATTATTAGATAAGACAATATCAGATAATATAATAAATGGCAGATGGATAATTCTA
TTGAGTAAGTTCCTAAAATTAATTAAGCTTGCAGGTGACAATAACCTCAACAATCTGAGTGAATTATATTTTTTGTTCAG
AATATTTGGACACCCAATGGTAGATGAAAGACAAGCCATGGATGCTGTTAAAGTTAATTGCAACGAGACCAAATTTTACT
TGTTAAGTAGTTTGAGTATGTTAAGAGGAGCTTTTATATATAGAATTATAAAAGGGTTTGTAAATAATTACAACAGATGG
CCTACTTTAAGAAATGCCATTGTCTTACCCTTAAGATGGTTAACTTACTATAAACTAAACACTTATCCTTCCTTGTTGGA
ACTTACAGAAAGAGATTTGATTGTTCTATCAGGACTACGTTTCTATCGAGAGTTTCGGTTGCCTAAAAAAGTGGATCTTG
AAATGATCATAAATGATAAGGCTATATCACCTCCTAAAAATTTAATATGGACTAGTTTCCCTAGAAATTATATGCCGTCA
CACATACAAAATTATATAGAACATGAAAAATTAAAATTCTCTGATAGTGATAAATCAAGAAGAGTATTAGAGTATTATTT
AAGAGATAACAAATTCAATGAATGTGATTTACACAACTGTGTAGTTAATCAAAGTTATCTTAACAACCCGAATCATGTGG
TATCATTGACAGGCAAAGAAAGAGAACTCAGTGTAGGTAGAATGTTTGCAATGCAACCAGGAATGTTCAGACAAGTTCAA
ATATTAGCAGAGAAAATGATAGCAGAAAACATATTACAATTTTTCCCTGAAAGTCTTACAAGATATGGTGATCTAGAACT
ACAAAAAATATTAGAATTGAAAGCAGGAATAAGTAACAAATCAAATCGTTACAATGATAATTACAACAATTACATTAGTA
AGTGCTCTATCATCACAGATCTCAGCAAATTCAATCAAGCATTTCGATATGAAACATCATGTATTTGTAGTGATGTACTG
GATGAACTGCATGGTGTACAATCTCTATTTTCCTGGGGACATTTAACTATTCCTCCTGTCACAATAATATGCACATATAG

GCATGCACCCCCCTATATAAAGGATCATATTGTAGATCTTAACAATGTAGATGAGCAAAGTGGACTATATAGATATCATA
TGGGTGGTATTGAAGGGTGGTGTCAAAAACTATGGACCATAGAAGCTATATCACTATTAGATCTAATATCTCTCAAAGGG
AAATTCTCAATTACTGCTTTAATTAATGGTGACAATCAATCAATAGATATAAGTAAACCAGTCAGACTCATGGAAGGTCA
AACTCATGCTCAAGCAGATTATATGCTAGCATTAAATAGTCTCAAATTACTGTATAAAGAGTATGCAGGAATAGGCCACA
AATTAAAAGGAACTGAGACTTATATATCGAGAGATATGCAATTTATGAGTAAAACGATCCAACATAACGGTGTATATTAC
CCAGCTAGTATAAAGAAAGTCCTAAGAGTGGGACCGTGGATAAACACTATACTTGATGACTTCAAAGTGAGTCTAGAATC
TATAGGTAGTTTGACACAAGAATTAGAATATAGAGGTGAAAGTCTATTATGCAGTTTAATATTTAGAAATGTATGGTTAT
ATAATCAAATTGCATTACAACTTAAAAATCATGCATTATGTAACAACAAATTATATTTGGATATATTAAAAGTTCTAAAA
CACTTAAAAACCTTTTTTAATCTTGATAACATTGATACAGCATTAACATTGTATATGAATTTGCCCATGTTATTTGGTGG
TGGTGATCCCAACTTGTTATATCGAAGTTTCTATAGAAGAACTCCTGATTTCCTCACAGAGGCTATAGTTCACTCTGTGT
TCATACTTAGTTATTATACAAACCATGATTTAAAAGATAAACTTCAAGATCTGTCAGATGATAGATTGAATAAGTTCTTA
ACATGCATAATCACGTTTGACAAAAACCCCAATGCTGAATTCGTTACATTGATGAGAGATCCTCAAGCTTTAGGATCTGA
GAGGCAAGCTAAAATTACTAGCGAAATCAATAGACTGGCAGTTACCGAGGTTTTGAGCACAGCTCCAAACAAAATATTTT
CCAAAAGTGCACAACACTATACCACTACAGAGATAGATCTTAATGATATTATGCAAAATATAGAACCTACATATCCTCAC
GGGCTAAGAGTTGTTTATGAAAGTTTACCCTTTTATAAAGCAGAGAAAATAGTAAATCTTATATCCGGTACAAAATCTAT
AACTAACATACTGGAAAAGACTTCTGCAATAGACTTAACAGATATTGATAGAGCCACTGAGATGATGAGGAAAAACATAA
CTTTGCTTATAAGGATATTACCATTAGATTGTAACAGAGATAAAAGAGAAATATTGAGTATGGAAAACCTAAGTATTACT
GAATTAAGCAAATACGTTAGAGAAAGATCTTGGTCTTTATCCAATATAGTTGGTGTTACATCACCCAGTATCATGTATAC
AATGGACATAAAATATACAACAAGCACTATAGCTAGTGGCATAATCATAGAGAAATATAATGTCAACAGTTTAACACGTG
GTGAGAGAGGACCCACTAAACCATGGGTTGGTTCATCTACACAAGAGAAAAAGACAATGCCAGTTTATAATAGACAAGTT
TTAACCAAAAAACAGAGAGATCAAATAGATCTATTAGCAAAATTGGATTGGGTGTATGCATCTATAGATAACAAGGATGA
ATTTATGGAGGAACTTAGCATAGGAACTCTTGGGTTAACATATGAGAAGGCCAAAAAATTATTCCCACAATATTTAAGTG
TTAACTATTTGCATCGTCTTACAGTCAGTAGTAGACCATGTGAATTCCCTGCATCTATACCAGCTTATAGAACTACAAAT
TATCACTTTGATACTAGCCCTATTAATCGCATATTAACAAAAAAGTATGGTGATGAAGATATTGATATAGTATTCCAAAA
CTGTATAAGCTTTGGCCTTAGCTTAATGTCTGTAGTAGAACAATTTACTAATGTATGTCCTAACAGAATTATTCTCATAC
CCAAGCTTAATGAGATACATTTGATGAAACCTCCCATATTCACAGGTGATGTTGATATTCACAAGTTAAAACAAGTGATA
CAAAAACAACATATGTTTTTACCAGACAAAATAAGTTTGACTCAATATGTGGAATTATTCTTAAGTAATAAAACACTCAA
ATCCGGATCTAATGTTAATTCTAATTTAATATTGGCGCATAAGATATCTGACTATTTTCATAATACTTACATTTTAAGTA
CTAATTTAGCTGGACATTGGATTCTTATTATACAACTTATGAAAGATTCTAAGGGTATTTTTGAAAAAGATTGGGGAGAG
GGATATATAACTGATCATATGTTCATTAATTTGAAAGTTTTCTTCAATGCTTATAAGACATATCTCTTGTGTTTTCATAA
AGGTTACGGCAGAGCAAAGCTGGAGTGTGATATGAATACTTCAGATCTCCTATGTGTATTGGAATTAATAGACAGTAGTT
ATTGGAAGTCTATGTCTAAGGTGTTTTTAGAACAAAAAGTTATCAAATACATTCTTAGCCAAGATGCAAGTTTACATAGA
GTAAAAGGATGTCATAGCTTCAAACTATGGTTTCTTAAACGTCTTAATGTAGCAGAATTCACAGTTTGCCCTTGGGTTGT
TAACATAGATTATCATCCAACACATATGAAAGCAATATTAACTTATATTGATCTTGTTAGAATGGGATTGATAAATATAG
ATAGAATATACATTAAAAATAAACACAAGTTCAATGATGAGTTTTATACTTCTAATCTGTTTTACATTAATTATAACTTC
TCAGATAATACTCATCTATTAACTAAACATATAAGGATTGCTAATTCCGAATTAGAAAGTAATTACAACAAATTATATCA
TCCCACACCAGAAACCCTAGAAAATATACTAACCAATCCGGTTAAAAGTAATGGAAAAAAGACACTGAGTGACTATTGTA
TAGGTAAAAATGTTGACTCAATAATGTTACCATCGTTATCTAATAAGAAGCTTATTAAATCGTCTACAATGATTAGAACC
AATTACAGCAGACAAGATTTGTATAATTTATTTCCTACGGTTGTGATTGATAAAATTATAGATCATTCAGGTAATACAGC
CAAATCTAACCAACTTTACACTACTACTTCTCATCAAATATCCTTAGTGCACAATAGCACATCACTTTATTGCATGCTTC
CTTGGCATCATATTAATAGATTCAATTTTGTATTTAGTTCTACAGGTTGTAAAATTAGTATAGAGTATATTTTAAAAGAT
CTTAAAATTAAGGATCCTAATTGTATAGCATTCATAGGTGAAGGAGCAGGGAATTTATTATTGCGTACAGTAGTGGAACT
TCATCCTGATATAAGATATATTTACAGAAGTCTGAAAGATTGCAATGATCATAGTTTACCAATTGAGTTTTTAAGGCTGT
ACAATGGACATATCAACATTGATTATGGTGAAAATTTGACCATTCCTGCTACAGATGCAACCAACAACATTCATTGGTCT
TATTTACATATAAAGTTTGCTGAACCTATCAGTCTTTTTGTCTGTGATGCTGAATTGCCTGTAACAGTCAACTGGAGTAA
GATTATAATAGAGTGGAGCAAGCATGTAAGAAAATGCAAGTACTGTTCTTCAGTTAATAAATGTACATTAATAGTAAAAT
ATCATGCTCAAGATGATATCGATTTCAAATTAGACAACATAACTATATTAAAAACTTATGTATGCTTAGGCAGTAAGTTA
AAGGGATCTGAAGTTTACTTAGTCCTTACAATAGGTCCTGCAAATGTGTTCCCAGTATTTAATGTAGTACAAAATGCTAA
ATTGATACTATCAAGAACCAAAAATTTCATCATGCCTAAAAAAGCTGATAAAGAGTCTATTGATGCAAATATTAAGAGTT
TGATACCCTTTCTTTGTTACCCTATAACAAAAAAAGGAATTAATACTGCATTGTCTAAATTAAAGAGTGTTGTTAGTGGA
GATATACTATCATATTCTATAGCTGGACGTAATGAAGTTTTCAGCAATAAACTTATAAATCATAAGCATATGAACATCTT
AAAGTGGTTCAATCATGTTTTAAATTTCAGATCAACAGAATTAAACTATAATCATTTATATATGGTAGAATCTACTTATC
CTCATCTAAGTGAATTGTTAAACAGCTTGACAACCAATGAACTTAAAAAACTGATTAAAATCACAGGTAGTTTGTTATAC
AACTTTTATAATGAATAATGAGCAAAAATCTTATAACAAAAATAGCTACACACTAACATTGTATTCAATTATAGTTATTT
AAAATTAATAATTATATAATTTTTAATAACTTCTAGTGAACTAATCCTAAAATTATCATTTTGATCTAGGAAGAATAAGT

```
TTAAATCCAAATCTAATTGGTTTATATGTATATTAACTAAATTACGAGATATTAGTTTTTGACACTTTTTTTCTCGT
>rsv012
ACGCGAAAAAATGCGTACTACAAACTTGCACATTCGGAAAAAATGGGGCAAATAAGAATTTGATAAGTGCTATTTAAGTC
TAACCTTTTTAATCAGAAATGGGGTGCAATTCACTGAGCATGATAAAGGTTAGATTACAAAATTTGTTTGATAATGACGA
AGTAGCATTGTTAAAAATAACATGTTATACTGACAAATTAATTCTTCTGACTAATGCATTAGCCAAAGCAACAATACATA
CAATTAAATTAAACGGCATAGTTTTTATACATGTTATAACAAGCAGTGAAGTGTGCCCTGATAACAATATTGTAGTGAAA
TCTAACTTTACAACAATGCCAATATTACAAAATGGAGGATACATATGGGAATTGATTGAATTGACACACTGCTCTCAATT
AAATGGTCTAATAGATGATAATTGTGAAATCAAATTTTCTAAAAGACTAAGTGACTCAGTAATGACTGATTATATGAATC
AAATATCTGATTTACTTGGGCTTGATCTCCATTCATGAATTATGTTTAGTCTAATTCAATAGACATGTGTTTATTACCAT
TTTAGTTAATATAAAACCTCATCAAAGGGAAATGGGGCAAATAAACTCACCCAATCAATCAAACCATGAGCACTACAAAC
GACAACACCACCATGCAAAGATTGATGATCACAGACATGAGACCCCTGTCGATGGATTCAATAATAACATCTCTCACCAA
AGAAATCATCACACACAAATTCATATACTTGATAAACAATGAATGTATTGTAAGAAAACTCGATGAAAGACAAGCTACAT
TTACATTCCTAGTCAATTATGAGATGAAGCTATTGCACAAAGTAGGGAGTACCAAATACAAGAAATACACCGAATATAAT
ACAAAATATGGCACATTCCCTATGCCTATATTTATCAATCATGGCGGGTTTCTAGAATGTATTGGCATCAAGCCTACAAA
ACATACTCCTATAATATACAAATATGACCTCAACCCGTAACTTCCAACAAAAAAACCAACTCATCCAAACCAAACCATTC
TCCAAACAACAATGCTCAACAGTTAAGAAGGAGCTAATCCATTTTAGTAATTAAAAATAAGGGTGAAACCAGTAACATAA
ATTGGGGCAAATACAAAGATGGCTCTTAGCAAAGTCAAGTTGAATGATACATTAAATAAGGATCAGCTGCTGTCATCCAG
CAAATACACTATTCAACGTAGTACAGGAGATAATATTGACACTCCCAATTATGATGTGCAAAAACACCTAAACAAACTAT
GTGGTATGCTATTAATCACTGAAGATGCAAATCATAAATTCACAGGATTAGTAGGTATGCTATATGCTATGTCCAGATTA
GGAAGGGAAGACACTATAAAGATACTTAAAGATGCTGGATATCATGTTAAAGCTAATGGAGTAGATATAACAACATATCG
TCAAGATATAAATGGAAAGGAAATGAAATTCGAAGTATTAACATTATCAAGCTTGACATCAGAAATACAAGTCAATATTG
AGATAGAATCTAGAAAGTCCTACAAAAAAATGCTAAAAGAGATGGGAGAAGTGGCTCCAGAATATAGGCATGATTCTCCA
GACTGTGGGATGATAATACTGTGTATAGCTGCCCTTGTAATAACCAAATTAGCAGCAGGAGATAGATCAGGTCTTACAGC
AGTAATTAGGAGGGCAAACAATGTCTTAAAAAACGAAATAAAACGCTACAAGGGCCTAATACCAAAAGACATAGCCAACA
GTTTTTATGAAGTGTTTGAAAAATACCCTCATCTTATAGATGTTTTTGTGCATTTTGGCATAGCACAATCATCCACAAGA
GGGGGCAGTAGAGTTGAAGGAATCTTTGCAGGATTGTTTATGAATGCCTATGGTTCAGGACAAGTAATGCTAAGATGGGG
AGTTTTAGCCAAATCTGTAAAAAATATCATGCTAGGACATGCTAGTGTCCAAGCAGAAATGGAGCAAGTTGTGGAAGTCT
ATGAGTATGCACAGAAGTTGGGAGGAGAAGCTGGTTTCTACCATATATTGAACAATCCAAAAGCATCATTGCTGTCATTA
ACTCAATTTCCTAACTTCTCAAGTGTGGTCCTAGGCAATGCAGCAGGTCTAGGCATAATGGGAGAGTATAGAGGTACACC
AAGAAACCAAGATCTCTATGATGCAGCCAAAGCATATGCAGAGCAACTCAAAGAAAATGGAGTAATAAACTACAGTGTAT
TAGACTTAACAACAGAAGAATTGGAAGCCATAAAGCATCAACTCAACCCCAAAGAAGATGACGTAGAGCTTTAAGTTAAC
AAAAAATACGGGGCAAATAAGTCAACATGGAGAAGTTTGCACCTGAATTTCATGGAGAAGATGCAAATAACAAAGCTACC
AAATTCCTAGAATCAATAAAAGGCAAGTTTGCATCATCCAAAGATCCTAAGAAGAAAGATAGCATAATATCTGTCAACTC
AATAGACATAGAAGTCACTAAAGAGAGCCCGATAACATCTGGCACCAACATTATCAATCCAACAAGTGACGCCGACAGTA
CCCCAGAAACTAAAGCCAACTACCCAAGAAAACCCCTAGTAAGCTTCAAAGAAGATCTCACCCCAAGTGATAACCCTTTC
TCTAAGTTGTACAAAGAAACCATAGAAACATTTGATAACAATGAAGAAGAATCTAGCTACTCATATGAGGAGATCAATGA
CCAAACAAATGACAACATTACAGCAAGACTAGATAGAATTGATGAAAAATTAAGTGAAATATTAGGAATGCTCCATACAT
TAGTAGTTGCAAGTGCAGGACCTACTTCGGCTCGTGACGGAATAAGAGATGCTATGGTTGGTCTAAGAGAAGAAATGATA
GAAAAAATAAGAGCAGAAGCATTAATGACCAATGATAGGTTAGAGGCTATGGCAAGACTTAGGAATGAGGAAAGCGAAAA
AATGGCAAAAGACACCTCAGATGAAGTGTCTCTCAATCCAACCTCCAAAAAATTGAGTGACTTGTTGGAAGACAACGATA
GCGACAATGATCTATCACTTGATGATTTTTGATCAGTGATCAACTCACTCAGCAATCAACAACATCAATAAAACAGACAC
CAATCCATTGAATCAATTGCCAGACTGAAAAAACAAACATCCATCAGCAGAACCACCAGCCAATCAATCAACCAATTGAT
CAATCAGCACCCTGACAAAATTAACAATATAGTAACAAAAAAAGAACAAGATGGGGCAAATATGGAAACATACGTGAACA
AGCTTCACGAAGGCTCCACATACACAGCAGCTGTCCAGTACAATGTTCTAGAAAAAGATGATGATCCCGCATCACTAACA
ATATGGGTGCCTATGTTCCAGTCATCTGTGCCAGCAGACTTGCTCATAAAAGAACTTGCAAGCATCAACATACTAGTAAA
GCAGATCTCTACGCCCAAAGGACCTTCACTACGAGTCACGATCAACTCAAGAAGTGCTGTGCTGGCTCAAATGCCTAGTA
ATTTCACCATAAGTGCAAATGTATCATTAGATGAAAGAAGCAAATTAGCATATGATGTAACTACACCTTGTGAAATCAAA
GCATGCAGTTTAACATGCTTAAAAGTAAAAAGTATGTTAACTACAGTCAAAGATCTAACCATGAAGACATTCAACCCCAC
TCATGAGATCATTGCTCTATGTGAATTTGAAAATATTATGACATCAAAAAGAGTAATAATACCAACCTATCTAAGATCAA
TTAGTGTCAAAAACAAGGATCTGAACTCACTGGAAAATATAGCAACCACCGAATTCAAAAATGCTATCACCAATGCTAAA
ATTATTCCCTATGCAGGATTAGTGTTAGTTATCACGGTTACTGATAATAAAGGAGCATTCAAGTATATCAAGCCACAGAG
TCAATTTATAGTGGATCTTGGTGCCTACCTAGAAAAAGAGAGCATATATTATGTGACTACTAATTGGAAGCATACAGCTA
CACGTTTTTCAATCAAACCACTAGAGGATTAAACTCAATTATCAACATTGAATGACAGGTTCACATATGTCCTCAACTGC
ACACTATATCTAAACATCATAAACATCTACACTACACACTTCATCACACAAACCAATCCCACTCAAAATCTAAAATCACT
TCCAGCCATTGTCTGCCAGACCTAGAGTGCGAATAGGTAAATAAAACAAGAATATGGGGTAAATAGATATCAGTTAGAGT
```

TCAACCAATCTCAACAACCATCTATACCGCCAATCCAATACATACATTGCAAATCTTAAAATGGGAAACACATCCATCAC
AATAGAATTCACAAGCAAATTTTGGCCCTATTTTACACTAATACATATGATCTTAACTCTAATCTCTTTACTAATTATAA
TCACTATTATGATTGCAATACTAAATAAGCTAAGTGAACATAAAATATTCTGCAACAAAACTCTTGAACAAGGACAGATG
TATCAAATCGACACATAGTGTTCTCCCATCATGCTGTATCAAATTATAATCTTGTGTATGTAAATAAACAAATCCAATCT
TCTCACAGAGTCATGGCATCACAAAACCATGCCAACCATCATGGTAGCATAGAGTAGTTANTTAAAAATTAACATAATGA
TGAATTATTAGTATGGGATCAAAAACAACATTGGGGCAAATGCAACAATGTCCAAAAACAAGAATCAACGCACTGCCAGG
ACTCTAGAAAAGACCTGGGATACTCTTAATCATCTAATTGTAATATCCTCTTGTTTATACAAATTAAATTTAAAATCTAT
AGCACAAATAGCACTATCAGTTTTGGCAATGATAATCTCAACCTCTCTTATAATTGCAGCCATAATATTCATCATCTCTG
CCAATCACAAAGTTACACTAACAACTGTCACAGTTCAAACAATAAAAAACCACACTGAGAAAAACATAACCACTTACCTT
ACTCAAGTCTCACCAGAAAGGGTTAGCCCATCCAAACAACCCACAGCTACACCACCAATCCACACAAACTCAGCCACAAT
ATCACCCAATACAAAATCAGAAACACACCATACAACAGCACAAACCAAAGGCACAACCTCTACTCCAACACAGAACAACA
AGCCAAGCACAAAACCACGTCCAAAAAATCCACCAAAAAAAGATGATTACCATTTTGAAGTGTTCAACTTTGTTCCCTGT
AGTATATGTGGCAACAATCAGCTCTGCAAATCCATTTGCAAAACAATACCAAGCAATAAACCAAAGAAAAAACCAACTAC
AAAACCTACAAACAAACCACCTACCAAAACCACAAACAAAAGAGACCCCAAAACACTAGCCAAACCACCGAAAAAAGAAA
CCACCATTAACCCAACAAAAAAACCAACCCCCAAGACTACAGAAAGAGACACCAACACCCCACAATCCACTGTGCTCGAC
ATAACCACATCAAAACACACAGAAAGGGACACCAGCACCTCACAATCCATTGCGCTTGACACAACCACATCAAAACACAC
AACCCAACAGCAATCTCTCTACTCAACCACCCCCGAAAACACACCCAACTCCACACAAACACCCACAGCATCCGAGCCCT
CCACATCAAATTCTACCTAAAGACTCCAGTCATATGCTTAGTTATTTAAAAACTACATCTTAGCAGAGAACCGTGATCCC
TCAAGCAAGAACGAAATTAAATCTGGGGCAAATAACCATGGAGTTGCTGATCCATAGATCAAGTGCAATCTTCCTAACTC
TTGCTATTAATGCATTGTACCTTACCTCAAGTCAGAACATAACTGAGGAGTTTTACCAATCAACATGTAGTGCAGTTAGC
AGAGGTTACTTGAGTGCTTTAAGAACAGGTTGGTATACCAGTGTCATAACAATAGAATTAAGTAATATAAAAGAAACCAA
ATGCAATGGAACTGACACTAAAGTAAAACTTATAAAACAAGAATTAGATAAGTATAAGAATGCAGTAACTGAATTACAGT
TACTTATGCAAAACACACCAGCTGTCAACAACCGGGCCAGAAGAGAAGCACCACAGTATATGAACTACACAATCAATACC
ACTAAAAACCTAAATGTATCAATAAGCAAGAAGAGGAAACGAAGATTTCTGGGCTTCTTGTTAGGTGTAGGATCTGCAAT
AGCAAGTGGTATAGCTGTATCCAAAGTTCTACACCTTGAAGGAGAAGTGAACAAGATCAAAAATGCTTTGCAGCTTACAA
ACAAAGCTGTAGTCAGTCTATCAAATGGGGTCAGTGTTTTAACCAGCAAAGTGTTAGATCTCAAGAATTATATAAACAAC
CAATTATTACCTATAGTAAATCAACAGAGTTGTCGCATATCCAACATTGAAACAGTTATAGAATTCCAGCAGAAGAACAG
CAGATTGTTGGAAATCACCAGAGAATTTAGTGTCAATGCAGGTGTAACGACACCTTTAAGCACTTACATGTTAACAAACA
GTGAGTTACTATCATTAATCAATGATATGCCTATAACAAATGATCAGAAAAAATTAATGTCAAGCAATGTTCAGATAGTA
AGGCAACAAAGTTATTCTATCATGTCTATAATAAAGGAAGAAGTCCTTGCATATGTTGTACAGCTACCTATCTATGGTGT
AATTGATACACCTTGCTGGAAATTACACACATCACCTCTGTGCACCACCAACATCAAAGAAGGATCAAATATTTGTTTAA
CAAGGACTGATAGAGGATGGTATTGTGATAATGCAGGATCAGTATCCTTCTTTCCACAAGCTGACACTTGTAAAGTACAG
TCCAATCGAGTATTTTGTGACACTATGAACAGTTTGACATTACCAAGTGAAGTCAGCCTTTGTAACACTGACATATTCAA
TTCCAAGTATGACTGCAAAATTATGACATCAAAAACAGACATAAGCAGCTCAGTAATTACTTCTCTAGGAGCTATAGTGT
CATGCTATGGTAAGACTAAATGTACTGCATCCAACAAAAATCGTGGAATTATAAAGACATTTTCTAATGGTTGTGATTAT
GTGTCAAACAAAGGAGTAGATACTGTATCAGTGGGCAACACTTTATATTATGTCAACAAGCTGGAAGGCAAAAACCTTTA
TGTAAAAGGGGAACCTATAATAAATTACTATGACCCTCTAGTGTTTCCTTCTGATGAGTTTGATGCATCAATATCTCAAG
TCAATGAAAAAATTAATCAAAGTTTAGCTTTTATTCGTAGATCCGATGAATTATTACATAATGTAAATACTGGAAAATCT
ACTACAAATATTATGATAACTGCAATTATTATAGTAATCATTGTAGTATTGTTATCATTAATAGCTATTGGTTTACTGTT
GTATTGCAAAGCCAAAAACACACCAGTTACATTAAGCAAAGACCAACTAAGTGGAATCAATAATATTGCATTCAGCAAAT
AGACAAAAAACCACCTGATCATGTTCCAACAACAATCTGCTGACCATCAATCCCAAATCAACTTACAACAGATACTTCAA
CATCACAGCACAGGCTGAATCATTTCCTCGCATCATGCTACCCACACAACTAAGCTAGATCCTTAATTCATAGTTACATA
AAAGCCTAAAATATCGCAATCAACACTAAATCAACACATCATTCACAAAACTAACAGCTGGGGCAAATATGTCGCGAAGA
AATCCCTGCAAATTTGAGATTAGAGGTCATTGCTTGAATGGTAGAAGATGCCACTACAGTCATAATTACTTTGAATGGCC
TCCTCATGCATTGCTAGTGAGGCAAAACTTCATGTTAAACAAGATACTCAAGTCAATGGACAAGAGCATAGACACTTTGT
CTGAAATAAGTGGAGCTGCTGAACTTGATAGAACAGAAGAATATGCTCTTGGTATAGTTGGAGTGCTAGAGAGTTACATA
GGATCTATAAACAACATAACAAAACAATCAGCATGTGTTGCTATGAGTAAACTTCTTATTGAGATCAATAGTGATGACAT
TAAAAAGCTGAGAGATAATGAAGAACCCAATTCACCTAAGATAAGAGTGTACAATACTGTTATATCATACATCGAGAGCA
ATAGAAAAAACAGCAAGCAAACCATCCATCTGCTTAAACGATTACCAGCAGACGTGCTGAAGAAGACAATAAAGAACACA
TTGGATATCCACAAAAGCATAACCATAAGCAACCCAAAAGAGTCAACCATAAGTGATCAAAATGACCAAACCAAAAATAA
TGATATTACCGGATAAATATCCTTGTAGTATATCATCCATATTGATCTCAAGTGAAAGCATGATTGCTACATTCAATCAT
AAAGACATATTACAATTTAACCACAACCATTTAGATAACCACCAGTGTTTATTAAATCATATATTTGATGAAATTCATTG
GACACCTAAAAACTTATTAGATGCCACTCAACAATTTCTCCAACATCTTAACATCCCTGAAGATATGTATACAGTATATA
TATTAGTGTCATAATGCTTGATCATAACGATTCTATATCATCCTACCATAAAACTGTCTTAATAAAGTTATGGGACAAAA
TGGATCCCATTATTAATGGAAGCTCTGCTAATGTATATCTAACTGATAGTTATCTAAAAGGTGTTATCTCTTTTTCAGAA

TGTAATGCTTTAGGGAGTTACCTTTTTAACGGCCCTTATCTTAAAAATGATTATACCAACTTAATTAGTAGACAAAGCCC
ACTAATAGAGCATATGAATCTAAAAAAACTAACTATAACACAATCATTAATATCTAGATACCATAAAGGTGAACTGAAAT
TAGAAGAACCAACTTATTTCCAGTCATTACTTATGACATATAAAAGCATGTCCTCGTCTGAACAAATTGCTACAACTAAC
TTACTTAAAAAAATAATACGAAGAGCTATAGAAATAAGTGATGTAAAGGTGTACGCCATCTTGAATAAACTAGGACTAAA
GGAAAAGGACAGAGTTAAGCCCAACAACAATTCAGGTGATGAAAACTCAGTACTTACAACCATAATTAAAGATGATATAC
TCTCAGCTGTGGAAAACAATCAATCATATACAAATTCAGATAAAAATTACTCAGTAAATCAAAATATCAATATCAAAACA
ACACTCTTAAAAAAGTTGATGTGTTCAATGCAACATCCTCCATCATGGTTAATACACTGGTTCAATTTATATACAAAATT
AAATAACATATTAACACAATATCGATCAAATGAGGTAAAAAGTCATGGGTTTATATTAATAGATAATCAAACTTTGAGTG
GTTTTCAGTTTATTTTAAATCAATATGGTTGCATTGTTTATCATAAAGGGCTCAAAAAAATTACAACTACTACATACAAT
CAATTTTTGACATGGAAAGACATCAGCCTCAGCAGATTAAATGTTTGCTTAATTACTTGGATAAGTAATTGTTTAAATAC
ATTAAATAAAAGCTTAGGGTTGAGATGCGGATTCAATAATGTTGTGCTATCACAATTATTCCTTTACGGAGATTGTATAC
TGAAATTATTTCATAATGAAGGCTTTTACATAATAAAAGAAGTAGAAGGATTTATTATGTCTTTAATTCTAAACATAACA
GAAGAAGATCAATTTAGGAAACGATTTTATAATAGCATGCTAAATAACATCACAGATGCAGCTATTAAGGCTCAAAAGGA
TCTACTATCAAGAGTATGTCACACTTTATTAGACAAGACAGTGTCTGATAATATCATAAATGGTAAATGGATAATTCTAT
TAAGTAAATTTCTTAAATTGATTAAGCTTGCAGGTGATAATAATCTCAATAACTTGAGTGAGCTATATTTTCTCTTCAGA
ATCTTTGGACATCCAATGGTTGATGAAAGACAAGCAATGGATGCTGTAAGAATTAACTGCAATGAAACTAAGTTCTATTT
ATTAAGTAGCCTAAGTACGTTGAGAGGTGCTTTCATTTATAGAATCATAAAAGGGTTTGTAAATACCTACAACAGATGGC
CCACTTTAAGGAATGCTATTGTCCTACCTCTAAGATGGTTAAACTATTATAAACTTAATACTTATCCATCTCTACTTGAA
ATCACAGAAAATGATTTGATTATTTTATCAGGATTGCGGTTCTATCGTGAGTTTCATCTGCCTAAAAAAGTGGATCTTGA
AATGATAATAAATGACAAAGCTATTTCTCCTCCAAAAGATCTAATATGGACTAGTTTTCCTAGAAATTACATGCCATCAC
ATATACAAAATTATATAGAACATGAAAAGTTGAAGTTCTCTGAAAGCGACAGATCAAGAAGAGTACTAGAGTATTACTTG
AGAGATAATAAATTCAATGAATGTGATCTATACAATTGTGTAGTTAATCAAAGCTATCTCAACAACTCTAATCATGTGGT
ATCACTAACTGGTAAAGAAAGAGAGCTCAGTGTGGGTAGAATGTTTGCTATGCAACCAGGTATGTTTAGGCAAATCCAAA
TCTTAGCAGAAAAAATGATAGCCGAAAATATTTTACAATTCTTCCCTGAGAGTTTGACAAGATATGGTGATCTAGAGCTT
CAAAAGATATTAGAATTAAAAGCAGGAATAAGCAACAAGTCAAATCGTTATAATGATAACTACAACAATTATATCAGTAA
ATGTTCTATAATAACAGATCTTAGCAAATTTAATCAAGCATTTAGATATGAAACATCATGTGTCTGCAGTGATGTATTAG
ATGAACTGCATGGGGTACAATCTCTATTCTCTTGGTTGCATTTAACAATACCTCTTGTCACAATAATATGTACATATAGA
CATGCACCTCCTTTTATAAAGGATCATGTTGTCAATCTTAATGAAGTTGATGAACAAAGTGGGTTATACAGATATCATAT
GGGTGGTATTGAGGGCTGGTGTCAAAAACTGTGGACCATTGAAGCCATATCATTATTAGATCTAATATCTCTTAAAGGTA
AATTCTCCATCACAGCTCTGATAAATGGTGATAATCAGTCAATTGATATAAGTAAACCAGTTAGACTTATAGAGGGTCAG
ACCCATGCTCAAGCAGATTATTTGTTAGCATTAAATAGCCTTAAATTGCTATATAAAGAGTATGCAGGCATAGGCCATAA
GCTTAAGGGAACTGAGACCTATATATCTCGAGATATGCAGTTCATGAGCAAAACAATCCAGCACAATGGAGTGTACTATC
CAGCCAGTATCAAAAAAGTCCTGAGAGTAGGTCCATGGATAAATACAATACTTGATGATTTTAAAGTTAGTTTAGAATCT
ATAGGTAGCTTAACACAGGAGTTAGAATACAGAGGAGAAAGCTTATTATGCAGTTTAATATTTAGGAATATTTGGTTATA
CAATCAAATTGCTCTGCAACTCCGAAATCATGCTTTATGTAATAATAAGCTATATTTAGATATATTGAAAGTATTAAAAC
ACTTAAAAACCTTTTTTAATCTTGATAGTATCGATACGGCGTTATCATTGTATATGAACTTGCCTATGCTGTTTGGTGGT
GGTGATCCTAATTTGTTATATCGAAGCTTTTATAGGAGAACTCCAGACTTCCTTACAGAAGCTATAGTACATTCAGTGTT
TGTGTTGAGCTATTATACTGGTCACGATCTACAAGATAAGCTCCAGGATCTTCCAGATGATAGACTGAACAAATTCTTGA
CTTGTGTCATCACATTTGATAAAAATCCAAATGCCGAGTTTGTAACATTGATGAGGGATCCACAGGCTTTAGGGTCTGAA
AGGCAAGCTAAAATTACTAGTGAGATTAATAGATTAGCAGTAACAGAAGTCTTAAGTATAGCTCCAAACAAAATATTTTC
TAAAAGTGCGCAACACTATACTACCACTGAGATTGATCTAAATGACATTATGCAAAATATAGAACCAACTTACCCTCATG
GATTAAGAGTTGTTTATGAAAGTTTACCTTTTTATAAAGCAGAAAAAATAGTTAATCTTATATCAGGAACAAAATCCATA
ACTAATATACTTGAAAAAACATCAGCGATAGATACAACTGATATTAATAGGGCTACTGATATGATGAGGAAAAATATAAC
CTTACTTATAAGGATACTTCCACTAGATTGTAACAAAGACAAAAGAGAGTTATTAAGTTTAGAAAATCTTAGCATAACTG
AATTAAGCAAGTATGTGAGAGAAAGATCTTGGTCATTATCCAATATAGTAGGAGTAACATCACCAAGTATTATGTTCACA
ATGGACATTAAATATACAACTAGCACTATAGCCAGTGGTATAATTATAGAAAAATATAATGTTAATGGTTTAACTCGTGG
TGAAAGAGGACCTACTAAGCCATGGGTAGGTTCATCTACACAGGAGAAAAAAACAATGCCAGTGTACAATAGACAAGTTT
TAACCAAAAAGCAAAGAGACCAAATAGATTTATTAGCAAAATTAGACTGGGTATATGCATCCATAGACAACAAAGATGAA
TTCATGGAAGAACTGAGTACTGGAACACTTGGACTGTCATATGAAAAGGCCAAAAAGTTGTTTCCACAATATCTAAGTGT
CAATTATTTACACCGGTTAACAGTCAGTAGTAGACCATGCGAATTCCCTGCCTCAATACCAGCTTATAGAACAACAAATT
ATCATTTTGATACTAGTCCTATCAATCATGTATTAACAGAAAAGTATGGAGATGAAGATATCGACATTGTGTTTCAAAAT
TGCATAAGTTTTGGTCTTAGCTTGATGTCAGTTGTGGAACAATTCACAAACATATGTCCTAATAGAATTATTCTCATACC
GAAGCTGAATGAGATACATTTGATGAAACCTCCTATATTTACAGGAGATGTTGATATCATCAAGTTGAAGCAAGTGATAC
AAAAACAGCATATGTTCCTACCAGATAAAATAAGTTTAACCCAATATGTAGAATTATTCTTAAGTAACAAAGCACTTAAA
TCTGGATCTCACATCAACTCTAATTTAATATTAGTACATAAAATGTCTGATTATTTTCATAATGCGTATATTTTAAGTAC

TAATTTAGCTGGACATTGGATTCTGATTATTCAACTTATGAAAGATTCAAAAGGTATTTTTGAAAAAGATTGGGGAGAGG
GGTATATAACTGATCATATGTTCATTAATTTAAATGTTTTCTTTAATGCTTATAAGACTTATTTGCTATGTTTTCATAGA
GGTTATGGTAAAGCAAAATTAGAATGTGATATGAACACTTCAGATCTTCTTTGTGTTTTGGAGTTAATAGACAGTAGCTA
CTGGAAATCTATGTCTAAAGTTTTCCTAGAACAAAAAGTCATAAAATACATAGTCAATCAAGACACAAGTTTGCATAGAA
TAAAAGGTTGTCACAGTTTTAAGTTGTGGTTTTTAAAACGCCTTAATAATGCTAAATTTACCGTATGCCCTTGGGTTGTT
AACATAGATTATCACCCAACACACATGAAAGCTATATTATCTTACATAGATTTAGTTAGAATGGGGTTAATAAATGTAGA
TAAATTAACCATTAAAAATAAAAACAAATTCAATGATGAATTTTACACATCAAATCTCTTTTACATTAGTTACAACTTTT
CAGACAACACTCATCTGCTAACAAAACAAATAAGGATTGCTAATTCAGAATTAGAAGATAATTATAACAAACTATATCAC
CCAACCCCAGAAGCTTTAGAAAATGTATCATTAATCCCTGTTAAAAGTAATAATAGAACCAAACCTAAATTTTGTATAAG
TGGAAGTACTGAATCTATGATGACGTCAACATTCTCTAATAAAATGCATATTAAATCTTCCACTGTTACCACAAGATTCA
ATTATAGCAGACAAGACTTGTACAATTTATTTCCAATTGTTGTGATAGACAGGATTATAGATCATTCAGGTAATACAGAA
AAATCTAACCAACTTTATACCACCACTTCACATCAGACATCTTTAGTAAGGAATAGTGCATCACTTTATTGCATGCTTCC
TTGGCATCATGTCAATAGATTTAACTTTGTATTTAGTTCCACAGGATGCAAGATCAGTATAGAATATATTTTAAAAGATC
TTAAGATTAAAGATCCCAGTTGTATAGCATTCATAGGTGAAGGAGCTGGTAACTTATTATTACGTACGGTAGTAGAACTT
CATCCTGACATAAGATATATTTACAGAAGTTTAAAAGATTGCAATGATCATAGTTTACCTATTGAATTTCTAAGGTTATA
CAACGGGCATATAAACATAGATTATGGTGAGAATTTAACCATTCCTGCTACAGATGCAACTAACAACATACATTGGTCTT
ATTTACATATAAAATTTGCAGAACCTATTAGTATCTTTGTCTGTGATGCTGAATTACCTGTCACAGCCAATTGGAGTAAA
ATCATAATTGAATGGAGTAAGCATGTAAGAAAGTGCAAATACTGTTCTTCTGTAAATAGATGCATTTTAATTGCAAAATA
CCATGCTCAAGATGATATTGATTTAAAATTAGATAACATTACTATATTAAAAACTTATGTGTGCCTAGGTAGCAAGTTAA
AAGGATCTGAAGTTTACTTAGTCCTTACAATAGGCCCTGCAAATATACTTCCTGTTTTTGATGTTGTGCAAAATGCTAAA
TTGATTCTTTCAAGAACTAAAAATTTCATTATGCCTAAAAAGATTGACAAGGAATCTATCGATGCAAATATTAAAAGCTT
AATACCTTTCCTTTGTTACCCTATAACAAAAAATGGAATTAAGACTTCATTGTCAAAATTGAAGAGTGTAGTTAATGGAG
ATATATTATCATATTCTATAGCTGGACGTAATGAAGTATTCAGCAACAAGCTTATAAACCACAAGCATATGAATATCTTA
AAATGGCTGGATCATGTTTTAAACTTTAGATCAGCTGAACTTAATTACAATCATTTATACATGATAGAGTCCACATATCC
TTACTTGAGTGAATTATTAAATAGTTTAACAACCAATGAGCTCAAGAAGCTGATTAAAATAACAGGTAGTGTACTATACA
ACCTTCCTAATGAACAGTAACTTAAAATATCATTAACAAGTTTGGTCAAATTTAGATGCTAACACATTATTATATTATAG
TTATTAAAAAATATGCAAACTTTTCAATAATTTAGCATATTGATTCCAAAATTATCATTTTAGTCTTAAGGGATTAAATA
AAAGTCTAAAACTAACAATCACACATGTGCATTTACAACACAACGAGACATTAGTTTTTGACACTTTTTTTCTCGT
>rsv013
TCAAATTAAATGGCATTGTATTTGTGCATGTTATTACAAGTAGTGATATTTGCCCTAATAATAATATTGTAGTGAAATCC
AATTTCACAACAATGCCAGTATTACAAAATGGAGGTTATATATGGGAAATGATGGAATTAACACACTGCTCTCAACCTAA
TGGCCTAATAGATGACAATTGTGAAATTAAATTCTCCAAAAAACTAAGTGATTCAACAATGACCAATTATATGAATCAAT
TATCTGAATTACTTGGATTTGACCTCAATCCATAAATCATAATAAATATCAACTAGCAAATCAATGTCACTAACACCATT
AGTTAATATAAAACTTGACAGAAGATAAAAATGGGGCAAATAAATCAATTCAGCCGACCCAACCATGGACACAACACACA
ATGATACCACACCACAAAGACTGATGATCACAGACATGAGACCATTATCACTAGAAACTATAATAACATCTCTGACCAGA
GATATCATAACACATAAATTTATATACTTGATAAATCATGAATGCATAGTGAGAAAACTTGATGAAAGACAGGCCACATT
TACATTTCTGGTCAACTATGAAATGAAACTATTGCACAAAGTGGGAAGCACTAAATATAAAAAATATACTGAATACAACA
CAAAATATGGCACTTTCCCTATGCCAATATTTATCAATCATGATGGGTTCTTAGAATGCATTGGCATTAAGCCTACCAAG
CACACACCCATAATATACAAGTATGATCTCAATCCATGAATATCAAAACAAGATTCAAACAATCCGAAATAACAACTTTA
TGCATAATCACACTCCATAGTCCAAATGGAGCCTGAAAATTATAGTTATTTAAAATTAAGGAGAGACATAAGATGAAAGA
TGGGGCAAATACAAAAATGGCTCTTAGCAAAGTCAAGTTGAATGATACACTCAACAAAGATCAACTTCTATCATCCAGCA
AATATACCATCCAACGGAGCACAGGAGACAGCATTGACACTCCTAATTATGATGTGCAGAAACACATTAATAAGTTATGT
GGCATGTTATTAATCACAGAAGATGCTAATCATAAATTCACTGGGTTAATAGGTATGTTATATGCTATGTCTAGATTAGG
AAGAGAAGACACCATAAAAATACTCAAAGATGCGGGATATCATGTTAAGGCAAATGGAGTGGATGTAACAACACATCGTC
AAGACATTAATGGGAAAGAAATGAAATTTGAAGTGTTAACATTAGCAAGCTTAACAACTGAAATTCAAATCAACATTGAG
ATAGAATCTAGAAAATCCTACAAAAAAATGCTAAAAGAAATGGGAGAGGTGGCTCCAGAATACAGGCATGACTCTCCTGA
TTGTGGGATGATAATATTATGTATAGCAGCATTAGTAATAACCAAATTAGCAGCAGGAGATAGATCTGGTCTTACAGCTG
TAATTAGGAGAGCTAATAATGTCCTAAAAAATGAAATGAAACGTTATAAAGGTTTATTACCCAAGGATATAGCCAACAGC
TTCTATGAAGTGTTTGAAAAATATCCTCACTTTATAGATGTTTTTGTTCATTTTGGTATAGCACAATCTTCTACCAGAGG
TGGCAGTAGAGTTGAAGGGATTTTTGCAGGATTGTTTATGAATGCCTATGGTGCAGGGCAAGTGATGTTACGGTGGGGG
TCTTAGCAAAATCAGTTAAAAACATTATGTTAGGACACGCTAGTGTACAAGCAGAAATGGAACAAGTTGTGGAGGTGTAT
GAGTATGCTCAGAAATTGGGTGGAGAAGCAGGATTCTACCATATATTGAACAACCCAAAAGCATCACTATTATCTTTGAC
TCAATTTCCTCACTTCTCTAGTGTAGTATTGGGCAATGCTGCTGGCCTAGGCATAATGGGAGAATACAGAGGTACACCAA
GGAATCAAGATTTATATGATGCTGCAAAAGCATATGCTGAACAACTCAAAGAAAATGGTGTGATTAACTACAGTGTATTA
GACTTGACAGCAGAAGAACTAGAGGCTATCAAACATCAGCTTAATCCAAAAGATAATGATGTAGAGCTTTGAGTTAATAA

AAAAATAAAAAAATGGGGCAAATAAATCATCATGGAAAAGTTTGCTCCTGAATTCCATGGAGAAGATGCAAATAACAGAG
CCACCAAATTCCTAGAATCAATAAAGGGCAAATTCACATCACCCAAAGATCCCAAGAAAAAAGATAGTATCATATCTGTC
AACTCAATAGATATAGAAGTAACCAAAGAAAGCCCTATAACATCAAATTCAACCATTATAAATCCAATAAATGAGACAGA
TGATACTATAGGGAACAAGCCCAATTATCAAAGAAAGCCTCTAGTAAGTTTCAAAGAAGACCCTACGCCAAGTGATAATC
CTTTTTCAAAACTATACAAAGAAACCATAGAAACATTTGATAACAATGAAGAAGAATCTAGCTATTCATATGAAGAAATA
AATGATCAGACAAACGATAATATAACAGCAAGATTAGATAGGATTGATGAGAAATTAAGTGAAATACTAGGAATGCTTCA
CACATTAGTAGTAGCGAGTGCAGGACCTACATCTGCTCGGGATGGTATAAGAGATGCCATGGTTGGTTTAAGAGAAGAAA
TGATAGAAAAAATCAGAACTGAAGCATTAATGACTAATGACAGACTAGAAGCTATGGCAAGACTCAGGAATGAAGAAAGT
GAAAAGATGGCAAAAGACACATCAGATGAAGTGTCTCTCAATCCAACATCAGAGAAACTGAACAACCTGTTGGAAGGGAA
TGACAGTGACAATGATCTATCACTTGAAGATTTCTGATTAGCTACCAAACTGTACATCAAAACACAACACCAATAGAAAA
CCAACAAACAAACCAACTCACCCATCCAACCAAACATCTATCTGCCGATTAGCCAACCAACCAAAAAACAACCAGCCAAT
CCAAAACTAGCCACCCGGAAAAAATCGATACTATAGTTACAAAAAAAGATGGGGCAAATATGGAAACATACGTGAACAAA
CTTCACGAGGGCTCCACATACACAGCTGCTGTTCAATACAATGTCCTAGAAAAAGACGATGATCCTGCATCACTTACAAT
ATGGGTGCCCATGTTCCAATCATCCATGCCAGCAGATCTACTCATAAAAGAACTAGCCAATGTCAATATACTAGTGAAAC
AAATATCCACACCCAAGGGACCCTCATTAAGAGTCATGATAAACTCAAGAAGTGCAGTGCTAGCACAAATGCCCAGCAAA
TTTACCATATGTGCCAATGTGTCCTTGGATGAAAGAAGCAAGCTGGCATATGATGTAACCACACCCTGTGAAATTAAGGC
ATGCAGTCTAACATGCCTAAAATCAAAAAATATGTTAACTACAGTTAAAGATCTCACTATGAAAACACTCAACCCAACAC
ATGACATCATTGCTTTATGTGAATTTGAAAATATAGTAACATCAAAAAAAGTCATAATACCAACATACCTAAGATCTATC
AGCGTCAGAAATAAAGATCTGAACACACTTGAAAATATAACAACCACTGAATTCAAAAATGCCATTACAAATGCAAAAAT
CATCCCTTACTCAGGATTACTGTTAGTCATCACAGTGACTGACAACAAAGGAGCATTCAAATATATAAAGCCACAAAGTC
AATTCATAGTAGATCTTGGAGCTTACCTTGAAAAAGAAAGTATATATTATGTTACAACAAATTGGAAGCACACAGCTACA
CGATTTGCAATCAAACCCATGGAAGATTAACCTTTTTCCTCTACATCAATGAGTAGATTCATACAAACTTTCTAACTACA
TTCTTCACTTCACAATCATAATCACCAACCCTCTGTGGTTCAATCAATCAAACAAAACTCATCAGGAGTTCCAGATCATC
CCAAGTCATTATTCATCAGATCCAGTACTCAAATAAGTTAATAAAAAATCCACATGGGGCAAATAATCATTGAGGGAAAT
CCAACTAATCACAACATCTGTCAACATAGACAAGTCAACACGCTAGATAAAATCAACCAATGGAAAATACATCCATAACT
ATAGAATTCTCAAGCAAATTCTGGCCTTACTTTACACTAATACACATGATAACAACAATAATCTCTTTGATAATCATAAT
CTCCATCATGATTGCAATACTAAACAAACTCTGCGAATATAATGTATTCCATAACAAAACCTTTGAGCTACCAAGAGCTC
GAGTCAATACATAGCATTCACCAATCTGATAGCTCAAAACAGTAACCTTGCATTTGTAAGTGAACTACCCTCACCTCTTC
ACAAAACCACATCAACATCTCACCATGCAAGCCATCATCTATACCATAAAGTAGTTAATTAAAAATAGTCATAACAATGA
ACTAGGATATTAAGACCAAAAACAACGCTGGGGCAAATGCAAACATGTCCAAAACCAAGGACCAACGCACCGCCAAGACA
CTAGAAAGGACCTGGGACACTCTCAATCATCTATTATTCATATCATCGTGCTTATACAAGTTAAATCTTAAATCTATAGC
ACAAATCACATTATCTATTTTGGCAATGATAATCTCAACCTCACTTATAATTGCAGCCATCATATTCATAGCTTCAGCAA
ACCACAAAGTCACACTAACAACTGCAATCATACAAGATGCAACGAACCAGATCAAGAACACAACCCCAACATACCTCACC
CAGAATCCCCAGCTTGGAATCAGCTTCTCCAATCTGTCCGGAACTACATCACAATCCACCACCATACTAGCTTCAACAAC
ACCAAGTGCTGAGTCAACCCCACAATCCACAACAGTCAAGATCAAAAACACAACAACAACCCAAATACTACCTAGCAAAC
CCACCACAAAACAACGCCAAAATAAACCACAAAACAAACCCAACAATGATTTTCACTTTGAAGTGTTCAATTTTGTACCC
TGCAGCATATGCAGCAACAATCCAACCTGCTGGGCCATCTGCAAGAGAATACCAAACAAAAAACCTGGAAAGAAAACCAC
CACCAAGCCCACAAAAAAACCAACCATCAAGACAACCAAAAAAGATCCCAAACCTCAAACCACAAAACCAAAGGAAGTAC
TCACCACCAAGCCTACAGAAAAGCCAACCATCGACACCACCAAAACAAACATCAGAACTACACTGCTCACCTCCAACACC
ACAGGAAATCCAGAACACACAAGTCAAGAGGAAACCCTCCACTCAACCACCTCCGAAGGCAATCTAAGCCCATCACAAGT
CTATACAACATCCGAGTACCTATCACAATCTCCATCTTCATCCAACACAACAAAATGATAGTCATTAAAAAGTGTATTGT
TGCAAAAAGCCATGACCAAATCAAACAGAATCAAAATCAACTCTGGGGCAAATAACAATGGAGTTGCCAATCCTCAAAAC
AAATGCTATTATCACAATCCTTGCTGCAGTCACACTCTGTTTCGCTTCCAGTCAAAACATCACTGAAGAATTTTATCAAT
CAACATGCAGTGCAGTTAGCAAAGGCTATCTTAGTGCTCTAAGAACTGGTTGGTATACTAGTGTTATAACTATAGAATTA
AGTAATATCAAGGAAAATAAGTGTAATGGTACAGACGCTAAGGTAAAATTAATAAAACAAGAATTAGATAAATATAAAAA
TGCTGTAACAGAATTGCAGTTGCTCATGCAAAGCACACCAGCAGCCAACAGTCGAGCCAGAAGAGAACTACCAAGATTTA
TGAATTATACACTCAACAATACCAAAAACACCAATGTAACATTAAGCAAGAAAAGGAAAAGAAGATTTCTTGGATTTTTG
TTAGGTGTTGGATCTGCAATCGCCAGTGGCATTGCCGTATCTAAGGTCCTGCACCTAGAAGGGGAAGTGAACAAAATCAA
AAGTGCTCTACTATCCACAAACAAGGCTGTAGTCAGCTTATCTAATGGAGTCAGTGTCTTAACCAGCAAAGTGTTAGACC
TCAAAAACTATATAGATAAACAGTTGTTACCTATTGTTAACAAGCAAAGCTGCAGCATATCAAACATTGAAACTGTGATA
GAGTTCCAACAAAAGAACAACAGACTACTAGAGATTACCAGAGAATTTAGTGTTAATGCAGGTGTAACTACACCTGTAAG
CACTTATATGTTAACTAATAGTGAGTTATTATCATTAATCAATGATATGCCTATAACAAATGATCAGAAAAAGTTAATGT
CCAGCAATGTTCAAATAGTTAGACAGCAAAGTTACTCTATCATGTCAATAATAAAAGAGGAAGTCTTAGCATATGTAGTA
CAATTACCACTATATGGTGTAATAGATACTCCTTGTTGGAAACTACACACATCCCCTCTATGTACAACCAACACAAAGGA
AGGATCCAACATCTGCTTAACAAGAACCGACAGAGGATGGTACTGTGACAATGCAGGATCAGTATCCTTTTTCCCACAAG

CTGAAACATGTAAAGTTCAATCGAATCGGGTATTTTGTGACACAATGAACAGTTTAACATTACCAAGTGAGGTAAATCTC
TGCAATATTGATATATTCAACCCCAAATATGATTGCAAAATTATGACTTCAAAGACAGATGTAAGCAGCTCCGTTATCAC
ATCTCTAGGAGCCATTGTGTCATGCTATGGCAAAACCAAATGTACAGCATCCAATAAAAATCGTGGGATCATAAAGACAT
TCTCTAACGGGTGTGATTATGTATCAAATAAGGGGGTGGATACTGTGTCTGTAGGTAATACATTATATTATGTAAATAAG
CAAGAAGGCAAAAGTCTCTATGTAAAAGGTGAACCAATAATAAATTTCTATGATCCATTAGTGTTCCCCTCTGATGAATT
TGATGCATCAATATCTCAAGTCAATGAGAAAATTAATCAGAGTCTAGCATTTATCCGTAAATCAGATGAATTATTACATA
ATGTAAATGCTGGTAAATCCACCACAAATATCATGATAACTACTATAATTATAGTAATTATAGTAATATTGTTAGCATTA
ATTGCAGTTGGACTGCTTCTATACTGCAAGGCCAGAAGCACACCAGTCACACTAAGTAAGGATCAACTGAGTGGTATAAA
TAATATTGCATTTAGTAACTGAATAAAAATAGCACCTAATCATATTCTTACAATGGTTCACTATTTGACCATAGATAACC
CATCTATCATTGGATTATCCTAAAATTTGAACTTCATCACAACTTTCATCTATAAACCATCTCACTTACACTATTTAAGT
AGATTCCTATTTTATAGTTATATAAAACAATTGAATACCAAATTAACTTACTATTTGTAAAAATGAGAACTGGGGCAAAT
ATGTCACGAAGGAATCCTTGCAAATTCGAAATTCGAGGTCATTGCTTGAATGGTAAAAGGTGTCATTTTAGTCATAATTA
TTTTGAATGGCCACCCCATGCACTACTTGTAAGACAAAACTTTATGTTAAACAGAATACTTAAGTCTATGGATAAAAGCA
TAGATACTTTGTCAGAAATAAGTGGAGCTGCAGAGTTGGACAGAACAGAAGAGTATGCCCTCGGTGTAGTTGGAGTGCTA
GAGAGTTATATAGGATCAATAAATAATATAACTAAACAATCAGCATGTGTTGCCATGAGCAAACTCCTCACTGAACTCAA
CAGCGATGACATCAAAAAACTAAGGGACAATGAAGAGCCAAACTCACCCAAAGTAAGAGTGTACAATACTGTCATATCAT
ATATTGAAAGCAACAGGAAGAACAATAAACAAACTATCCATCTGTTAAAAAGATTGCCAGCAGACGTATTGAAGAAAACC
ATCAAAAACACATTGGATATCCACAAGAGCATAACCATCAATAACCCAAAAGAATCAACTGTTAGTGATACGAATGACCA
TGCCAAAAATAATGATACTACCTGACAAATATCCTTGTAGTATAAATTCCATACTAATAACAAGTAATTGTAGAGTCACT
ATGTATAATCAAAAAAACACACTATATATCAATCAAAACAACCAAAATAACCATATATACCCACCGGATCAACCATTCAA
TGAAATCCATTGGACCTCTCAAGACTTGATTGATGCAACTCAAAATTTTCTACAACATCTAGGTATTACTGATGATATAT
ACACAATATATATATTAGTGTCATAATACTCAATCCTAATACTTACCACATCATCAAATTATTAACTCAAACAATTCAAG
CTATGGGACAAAATGGATCCCATTATTAGTGGAAATTCTGCTAATGTTTATCTAACTGATAGTTATTTAAAAGGTGTTAT
TTCTTTCTCAGAATGTAACGCCTTAGGTAGTTACATATTCAATGGTCCTTATCTCAAAAATGATTATACCAACTTAATTA
GTAGACAAAATCCATTAATAGAACACATAAATCTAAAGAAACTAAATATAACACAGTCCTTAATATCTAAGTATCATAAA
GGTGAAATAAAAATAGAAGAACCTACTTACTTTCAGTCATTACTTATGACATACAAGAGTATGACCTCGTCAGAACAGAC
TACTACTACTAATTTACTTAAAAAGATAATAAGAAGAGCTATAGAAATCAGTGATGTTAAAGTCTATGCTATATTGAATA
AACTGGGGCTCAAAGAAAAAGACAAGATTAAATCCAATAATGGACAAGATGAAGACAACTCAGTTATTACTACCATAATC
AAAGATGATATACTTTTAGCTGTCAAGGATAATCAATCTCATCTTAAAGCAGACAAAAATCAATCCACAAAACAAAAAGA
TACAATCAAAACAACACTTTTGAAGAAATTAATGTGTTCGATGCAACATCCTCCATCATGGTTAATACATTGGTTTAACT
TATACGCAAAATTAAACAGCATATTAACACAGTATCGATCTAGTGAGGTAAAAAACCATGGTTTTATATTGATAGATAAT
CATACTCTTAGTGGATTCCAATTTATTTTGAATCAATATGGTTGTATAGTTTATCATAAGGAACTCAAAAGAATTACTGT
GACAACTTATAATCAATTCTTGACATGGAAAGATATTAGCCTTAGTAGATTAAATGTTTGTTTGATTACATGGATTAGTA
ACTGTTTGAACACATTAAATAAAAGCTTAGGCTTAAGATGTGGATTCAATAATGTTATCTTGACACAATTATTCCTTTAT
GGAGATTGTATACTAAAACTATTCCACAATGAGGGGTTCTACATAATAAAAGAGGTAGAGGGGTTTATTATGTCTCTAAT
TTTAAATATAACAGAAGAAGATCAATTCAGAAAACGGTTTTATAATAGTATGCTCAACAACATCACAGATGCCGCTAATA
AAGCTCAGAAAAATCTGCTATCAAGAGTATGTCATACATTATTAGATAAGACAATATCAGATAATATAATAAATGGCAGA
TGGATAATTCTATTGAGTAAGTTCCTTAAATTAATTAAGCTTGCAGGTGACAATAACCTCAACAATCTGAGTGAATTATA
TTTTTTGTTCAGAATATTTGGACACCCAATGGTAGATGAAAGACAAGCCATGGATGCTGTTAAAGTTAATTGCAACGAGA
CCAAATTTTACTTGTTAAGTAGTTTGAGTATGTTAAGAGGAGCTTTTATATATAGAATTATAAAAGGGTTTGTAAATAAT
TACAACAGATGGCCTACTTTAAGAAATGCCATTGTTTTACCCTTAAGATGGTTAACTTACTATAAACTAAACACTTATCC
TTCCTTGTTGGAACTTACAGAAAGAGATTTGATTGTTCTATCAGGACTACGTTTCTATCGAGAGTTTCGGTTGCCTAAAA
AAGTGGATCTTGAAATGATCATAAATGATAAGGCTATATCACCTCCTAAAAATTTAATATGGACTAGTTTCCCTAGAAAT
TATATGCCGTCACACATACAAAATTATATAGAACATGAAAAATTAAAATTCTCTGATAGTGATAAATCAAGAAGAGTATT
AGAGTATTATTTAAGAGATAACAAATTCAATGAATGTGATTTATACAACTGTGTAGTTAATCAAAGTTATCTTAACAACC
CGAATCATGTGGTATCATTGACAGGCAAAGAAAGAGAACTCAGTGTAGGTAGAATGTTTGCAATGCAACCAGGAATGTTC
AGACAAGTTCAAATATTAGCAGAGAAAATGATAGCTGAAAACATATTACAATTTTTCCCTGAAAGTCTTACAAGATATGG
TGATCTAGAACTACAGAAAATATTAGAATTGAAAGCAGGAATAAGTAACAAATCAAATCGTTACAATGATAATTACAACA
ATTACATTAGTAAGTGCTCTATCATCACAGATCTCAGCAAATTCAATCAAGCATTTCGGTATGAAACATCATGTATTTGT
AGCGATGTACTGGATGAACTGCATGGTGTACAATCTCTATTTTCCTGGTTACATTTAACTATTCCTCATGTCACAATAAT
ATGCACATATAGGCATGCACCCCCCTATATAAAGGATCATATTGTAGATCTTAACAATGTAGATGAGCAAAGTGGATTAT
ATAGATATCATATGGGTGGTATCGAAGGGTGGTGTCAAAAACTATGGACCATAGAAGCTATATCACTATTAGATCTAATA
TCTCTCAAAGGGAAATTCTCAATTACTGCTTTAATTAATGGTGACAATCAATCAATAGATATAAGTAAACCAGTCAGACT
CATGGAAGGTCAAACTCATGCTCAAGCAGATTATTTGCTAGCATTAAATAGTCTTAAATTACTGTATAAAGAGTATGCAG
GAATAGGCCACAAATTAAAAGGAACTGAGACTTATATATCGAGAGATATGCAATTTATGAGTAAAACGATCCAACATAAC

GGTGTATATTACCCAGCTAGTATAAAGAAAGTCCTGAGAGTGGGACCGTGGATAAACACTATACTTGATGACTTCAAAGT
GAGTCTAGAATCTATAGGTAGTTTGACACAAGAATTAGAATATAGAGGTGAAAGTCTATTATGCAGTTTAATATTTAGAA
ATGTATGGTTATATAATCAAATTGCATTACAACTTAAAAATCATGCATTATGTAACAACAAATTATATTTGGACATATTA
AAAGTTCTAAAACACTTAAAAACCTTTTTTAATCTTGATAACATTGATACAGCATTAACATTATATATGAATTTGCCCAT
GTTATTTGGTGGTGGTGATCCCAACTTGTTATATCGAAGTTTCTATAGAAGAACTCCTGATTTCCTCACAGAGGCTATAG
TTCACTCTGTGTTCATACTTAGTTATTATACAAACCATGATTTAAAAGATAAACTTCAAGATCTGTCAGATGATAGATTG
AATAAGTTCTTAACATGCATAATCACGTTTGACAAAAACCCCAATGCTGAATTCGTTACATTGATGAGAGATCCTCAAGC
TTTAGGATCTGAGAGGCAAGCTAAAATTACTAGCGAAATCAATAGACTGGCAGTTACCGAGGTTTTGAGCACAGCTCCAA
ACAAAATATTCTCCAAAAGTGCACAACACTATACCACTACAGAGATAGATCTTAATGATATTATGCAAAATATAGAACCT
ACATATCCTCACGGGCTAAGAGTTGTTTATGAAAGTTTACCCTTTTATAAAGCAGAGAAAATAGTAAATCTTATATCCGG
TACAAAATCTATAACTAACATACTGGAAAAGACTTCTGCCATAGACTTAACAGATATTGATAGAGCCACTGAGATGATGA
GGAAAAACATAACTTTGCTTATAAGGATATTACCATTAGATTGTAACAGAGATAAAAGAGAAATATTGAGTATGGAAAAC
CTAAGTATTACTGAATTAAGCAAATACGTTAGAGAAAGATCTTGGTCTTTATCCAATATAGTTGGTGTTACATCACCCAG
TATCATGTATACAATGGACATAAAATATACAACAAGCACTATAGCTAGTGGCATAATCATAGAGAAATATAATGTCAACA
GTTTAACACGTGGTGAGAGAGGACCCACTAAACCATGGGTTGGTTCATCTACACAAGAGAAAAAGACAATGCCAGTTTAT
AATAGACAAGTTTTAACCAAAAAACAGAGAGATCAAATAGATCTATTAGCAAAATTGGATTGGGTGTATGCATCTATAGA
TAACAAGGATGAATTTATGGAGGAACTTAGCATAGGAACTCTTGGGTTAACATATGAGAAGGCCAAAAAATTATTTCCAC
AATATTTAAGTGTTAACTATTTGCATCGTCTTACAGTCAGTAGTAGACCATGTGAATTCCCTGCATCTATACCAGCTTAT
AGAACTACAAATTATCACTTTGATACTAGCCCTATTAATCGCATATTAACAGAAAAGTATGGTGATGAAGATATTGATAT
AGTATTCCAAAACTGTATAAGCTTTGGCCTTAGCTTAATGTCAGTAGTAGAACAATTTACTAATGTATGTCCTAACAGAA
TTATTCTCATACCCAAGCTTAATGAGATACATTTGATGAAACCTCCCATATTCACAGGTGATGTTGATATTCACAAGTTA
AAACAAGTGATACAAAAACAACATATGTTTTTACCAGACAAAATAAGTTTGACTCAATATGTGGAATTATTTTTAAGTAA
TAAAACACTCAAATCTGGATCTAATGTTAATTCTAATTTAATATTGGCGCATAAGATATCTGACTATTTTCATAATACTT
ACATTTTAAGTACTAATTTAGCTGGACATTGGATTCTTATTATACAACTTATGAAAGATTCTAAGGGTATTTTTGAAAAA
GATTGGGGAGAGGGATATATAACTGATCATATGTTCATTAATTTGAAAGTTTTCTTCAATGCTTATAAGACATATCTCTT
GTGTTTTCATAAAGGTTACGGCAGAGCAAAGCTGGAGTGTGATATGAATACTTCAGATCTCCTATGTGTATTGGAATTAA
TAGACAGTAGTTATTGGAAGTCTATGTCTAAGGTGTTTTTAGAACAAAAAGTTATCAAATACATTCTTAGCCAGGATGCA
AGTTTACATAGAGTAAAAGGATGTCATAGCTTCAAACTATGGTTTCTTAAACGTCTTAATGTAGCAGAATTCACAGTTTG
CCCTTGGGTTGTTAACATAGATTATCATCCAACACATATGAAAGCAATATTAACTTATATTGATCTTGTTAGAATGGGAT
TAATAAATATAGATAGAATATACATTAAAAATAAACACAAGTTCAATGATGAATTTTATACTTCTAATCTTTTTTACATT
AATTATAACTTCTCAGATAATACTCATCTATTAACTAAACATATAAGGATTGCTAATTCTGAATTAGAAAGTAATTACAA
CAAATTATATCATCCTACACCAGAAACCCTAGAAAATATACTAACCAATCCGGTTAAAAGTAATGATAAAAAGACACTGA
GTGACTATTGTATAGGTAAAAATGTTGACTCAATAATGTTACCATCGTTATCTAATAAGAAGCTTATTAAATCGTCTACA
ATGATTAGAACCAATTACAGCAGACAAGATTTGTATAATTTATTTCCTACGGTTGTGATTGATAAAATTATAGATCATTC
AGGTAATACAGCCAAATCTAACCAACTTTACACTACTACTTCTCATCAAATATCCTTAGTGCACAATAGCACATCACTTT
ATTGCATGCTTCCTTGGCATCATATTAATAGATTCAATTTTGTATTTAGTTCTACAGGTTGTAAAATTAGTATAGAGTAT
ATTTTAAAAGATCTTAAAATTAAGGATCCTAATTGTATAGCATTCATAGGTGAAGGAGCAGGGAATTTATTATTGCGTAC
AGTAGTGGAACTTCATCCTGATATAAGATATATTTACAGAAGTCTGAAAGATTGCAATGATCATAGTTTACCAATTGAGT
TTTTAAGGCTGTACAATGGGCATATCAACATTGATTATGGTGAAAATTTGACCATTCCTGCTACAGATGCAACCAACAAC
ATTCATTGGTCTTATTTACATATAAAGTTTGCTGAACCTATCAGTCTTTTTGTCTGTGATGCTGAATTGCCTGTAACAGT
CAACTGGAGTAAGATTATAATAGAGTGGAGCAAGCATGTAAGAAAATGCAAGTACTGTTCTTCAGTTAATAAATGTACAT
TAATAGTAAAATATCATGCTCAAGATGATATCGATTTCAAATTAGACAACATAACTATATTAAAAACTTATGTATGCTTA
GGCAGTAAGTTAAAGGGATCTGAAGTTTACTTAGTCCTTACAATAGGTCCTGCAAATGTGTTCCCAGTATTTAATGTAGT
ACAAAATGCTAAATTGATACTATCAAGAACCAAAAATTTCATCATGCCTAAAAAAGCTGATAAAGAGTCTATTGATGCAA
ATATTAAGAGTTTGATACCCTTTCTTTGTTACCCTATAACAAAAAAAGGAATTAATACTGCATTGTCTAAATTAAAGAGT
GTTGTTAGTGGAGATATACTATCATATTCTATAGCTGGACGTAATGAAGTTTTCAGCAATAAACTTATAAATCATAAGCA
TATGAACATCCTAAAGTGGTTCAATCATGTTTTAAATTTCAGATCAACAGAATTA
